# Plethysmography Phenotype QTL in Mice Before and After Allergen Sensitization and Challenge

**DOI:** 10.1534/g3.116.032912

**Published:** 2016-07-21

**Authors:** Samir N. P. Kelada

**Affiliations:** Department of Genetics, University of North Carolina, Chapel Hill, North Carolina 27599

**Keywords:** asthma, allergic airway disease, QTL

## Abstract

Allergic asthma is common airway disease that is characterized in part by enhanced airway constriction in response to nonspecific stimuli. Genome-wide association studies have identified multiple loci associated with asthma risk in humans, but these studies have not accounted for gene–environment interactions, which are thought to be important factors in asthma. To identify quantitative trait loci (QTL) that regulate responses to a common human allergen, we applied a house dust mite mouse (HDM) model of allergic airway disease (AAD) to 146 incipient lines of the Collaborative Cross (CC) and the CC founder strains. We employed a longitudinal study design in which mice were phenotyped for response to the bronchoconstrictor methacholine both before and after HDM sensitization and challenge using whole body plethysmography (WBP). There was significant variation in methacholine responsiveness due to both strain and HDM treatment, as reflected by changes in the WBP parameter enhanced pause. We also found that distinct QTL regulate baseline [chromosome (Chr) 18] and post-HDM (Chr 19) methacholine responsiveness and that post-HDM airway responsiveness was correlated with other features of AAD. Finally, using invasive measurements of airway mechanics, we tested whether the Chr 19 QTL affects lung resistance *per se* using C57BL/6J mice and a consomic strain but found that QTL haplotype did not affect lung resistance. We conclude that aspects of baseline and allergen-induced methacholine responsiveness are associated with genetic variation, and that robust detection of airway resistance QTL in genetically diverse mice will be facilitated by direct measurement of airway mechanics.

Allergic asthma is a common disease that affects ∼10–15% of the population and causes substantial decrements in quality of life and productivity (Mannino *et al.* 1998, 2002). The disease is characterized by elevated IgE, airway inflammation, mucus hyper-secretion, and airway hyper-responsiveness (Busse and Lemanske 2001). Asthma is thought to result from a combination of both genetic and environmental risk factors (von Mutius 2009; Ober and Vercelli 2012; Rava *et al.* 2015) and significant progress has been made toward the identification of both types of risk factors, particularly genetic risk factors as of late. Genome-wide association studies (GWAS) have identified at least 20 distinct loci with asthma in multiple study populations (Welter *et al.* 2014; Bønnelykke *et al.* 2015; Ortiz and Barnes 2015). Still, the percent of heritable and total disease risk explained remains low (Moffatt *et al.* 2010), and these studies have not accounted for gene–environment interactions.

Mouse models offer a tractable system to interrogate gene–environment interactions, and mouse models have already been used to identify genetic loci associated with lung function parameters that are relevant to asthma. A variety of phenotyping methods have been used to evaluate airway responsiveness to cholinergic agonists that induce bronchoconstriction and thus cause an increase in resistance to airflow. Quantitative trait locus (QTL) mapping studies using these phenotyping methods have identified loci on almost every chromosome in the mouse genome (Levitt and Mitzner 1988, 1989; De Sanctis *et al.* 1995, 1999; Ewart *et al.* 1996, 2000; Zhang *et al.* 1999; McIntire *et al.* 2001; Camateros *et al.* 2010; Leme *et al.* 2010). These results suggest that due to the use of different phenotyping approaches these studies have examined distinct phenotypes (Berndt *et al.* 2011) and/or that airway resistance is truly a complex trait with contributions from many loci. It is important to note that by and large these studies have used only classical inbred strains or derivatives thereof. Additionally, only three studies (Zhang *et al.* 1999; Ewart *et al.* 2000; McIntire *et al.* 2001) have examined airway phenotypes in the context of allergen-induced inflammation and thereby directly considered the possibility of gene–environment interactions.

To address the role of gene–environment interactions, we developed a house dust mite (HDM) model of allergic airway disease (AAD) (Kelada *et al.* 2011) and applied this model to incipient lines of the Collaborative Cross (CC). The CC is composed of a panel of recombinant inbred lines derived from eight-way crosses using five classical inbred strains (C57BL/6J, 129S1/SvImJ, A/J, NOD/ShiLtJ, and NZO/HlLtJ) and three wild-derived inbred strains (WSB/EiJ, PWK/PhJ, and CAST/EiJ) (Consortium 2012) whose genomes have recently been sequenced (Keane *et al.* 2011; Yalcin *et al.* 2011). Using incipient CC lines, which we refer to as “preCC” mice, we have identified QTL for relevant AAD phenotypes including eosinophilic (chr 11) (Kelada *et al.* 2014) and neutrophilic (Chrs 2,4,7) (Rutledge *et al.* 2014) inflammation, as well as gene expression in the lung (*i.e.*, eQTL) (Kelada *et al.* 2014).

Here we report on one feature of the physiologic response to HDM, namely bronchoconstrictor (methacholine) response as measured by whole body plethysmography (WBP). To evaluate the effect of HDM treatment in preCC mice (which were not inbred/clonal), we employed a longitudinal study design in which mice were phenotyped using WBP both before and after HDM treatment, allowing us to distinguish between methacholine response phenotypes attributable solely to genotype *vs.* genotype and HDM treatment. To examine CC founder strains in matched conditions, we used the same longitudinal study with multiple replicates per strain. We do not argue that the WBP parameter we examined, enhanced pause (Penh), represents airflow resistance *per se*, but rather employed plethysmography as a screening approach with the intent of subsequently validating results using invasive measurements of airflow resistance.

As expected based on prior studies (Zhang *et al.* 1997; Brewer *et al.* 1999; Whitehead *et al.* 2003; Van Hove *et al.* 2009; Leme *et al.* 2010; Kelada *et al.* 2011, 2014), we detected significant variation due to both strain and HDM treatment. We found that distinct loci regulate baseline (chromosome (Chr) 18) and post-HDM (Chr 19) methacholine responsiveness and that post-HDM methacholine responsiveness was correlated with other biomarkers of AAD. Finally, we evaluated whether the post-HDM methacholine responsiveness QTL had an effect on lung resistance in an independent set of experiments using HDM challenged C57BL/6J mice and a consomic strain.

## Materials and Methods

### Mice

We obtained 153 male preCC mice (ages 10–14 wk) from Oak Ridge National Laboratory (Chesler *et al.* 2008; Kelada *et al.* 2012, 2014; Rutledge *et al.* 2014). Each mouse was from an independent CC line that had undergone five to fourteen generations of inbreeding. For each of the eight CC founder strains, we obtained five to nine mice per strain. All mice were singly housed, with alpha-dri bedding, in the same facility under normal 12-hr light/dark cycles. Inbred strains, including C57BL/6J-Chr19^A^/NaJ were obtained from the Jackson Laboratory and were also singly housed to mimic preCC mice. All experiments with mice were conducted at a facility accredited by the Association for Assessment and Accreditation of Laboratory Animal Care and the protocol was approved by an institutional animal care and use committee protocol.

### Phenotyping protocol

**Figure 1:**
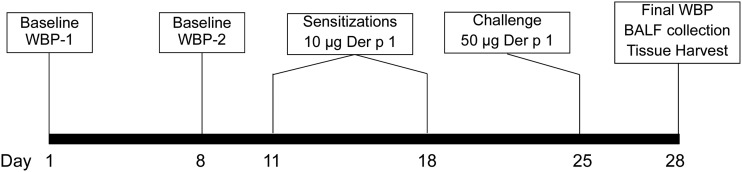
Study design featuring longitudinal WBP measurements.

The study design is shown in Figure 1 and all preCC and CC founder phenotype WBP data are provided in Supplemental Material, File S1. For these studies, we adapted an established protocol that produces hallmark features of AAD (Kelada *et al.* 2011). Since each preCC mouse was unique (*i.e.*, not yet fully inbred), we chose to evaluate effect of allergic inflammation on airway responsiveness to methacholine using a longitudinal study design in which WBP measurements were made before and after the induction of AAD by sensitization and challenge with HDM allergen. We note that longitudinal studies using plethysmography have been used before to evaluate changes in airway responsiveness to methacholine due to allergic inflammation (Lofgren *et al.* 2006). On day 1, dose–response measurements using methacholine (0, 3.1, 6.2, 12.5, and 25 mg/ml) were made (Figure 1). One week later, the same measurements were made to examine reproducibility, though in a minority of cases this was not feasible (*n* = 34 preCC mice). Mice were then sensitized with 10 µg (in 100 µl) of the immunodominant allergen from the *Dermatophagoides pteronyssinus* species of HDM, Der p 1 (Indoor Biotechnology, Cat. No. LNT-DP1-1), administered by intraperitoneal injection on days 11 and 18 of the protocol. On day 25, mice were challenged with 50 µg of Der p 1 (in 40 µl), administered by oro-pharyngeal aspiration. Mice were phenotyped by WBP for a final time on day 28. Immediately after the last WBP measurement, mice were sacrificed by an overdose of pentobarbitol, followed by collection of blood, whole lung lavage fluid, and lung tissue. During the course of WBP data collection, some mice exhibited responses to methacholine that necessitated removal from the WBP chamber prior to a subsequent (higher) dose of methacholine to prevent respiratory difficulty. The decision to remove the mouse from the WBP chamber was based on visual observation of the mouse and not on a particular threshold value of Penh because we found that some mice exhibited signs of respiratory difficulty at lower values of Penh compared to others. Removal of a mouse from the WBP chamber led to incomplete dose–response data. However, even with the truncated dose–response data, we were able to generate an overall phenotype of methacholine responsiveness that was used for QTL mapping, as described below.

### WBP data analysis

The analysis of WBP data was focused on the derived parameter (Penh). The two baseline measurements of Penh were found to be reproducible on a mouse-by-mouse basis, as shown in Figure S1. As such, the two sets of baseline WBP data were averaged together to create a mean value of Penh per dose of methacholine. For mice for which only one baseline measurement was available, the single measurement was used as the mean. We then used a simple modeling approach that is analogous to what is used in human studies where methacholine dose–response data are used to estimate the dose required to elicit a 20% reduction in forced expiratory volume in the first second. First, for each mouse, the value of Penh per methacholine dose was converted to a percent control value, *i.e.*, the ratio of Penh at a given dose of methacholine compared to Penh when methacholine equals 0, multiplied by 100. We then fit a quadratic model to the data (on a mouse-by-mouse basis):

$$\text{Penh}\left(\text{\%}\;\text{control}\right)=\alpha +\beta_{\text{1}}\left(\text{MCh}\right)+\beta_{\text{2}}\left({\text{MCh}}^{2}\right)+\text{error}$$

where MCh = methacholine (mg/ml). Example quadratic model results are shown in Figure S2. The quadratic model was used to calculate the dose of methacholine required to produce a 50% increase in baseline Penh, which we refer to as the provocative concentration 150 (PC_150_). In addition to mimicking the modeling approach in humans, this approach also allowed for the calculation of a phenotype value (PC_150_) for mice for which we had incomplete methacholine dose–response data. This is because mice that were removed from the WBP chambers prior to completing the full dose–response assessment tended to be very responsive to methacholine, and thus interpolating a value that produced a 50% increase in Penh (% control) was feasible. We note that the threshold we used, namely a 50% increase, is considered a reasonable value (Walker *et al.* 2013) but could be increased to achieve wider phenotype distributions but this comes at a cost of not being able to calculate a phenotype value (*e.g.*, PC_200_) for mice that never exhibited a doubling of Penh during the methacholine dose–response (see Figure S2). For one subject (OR3584m51), quadratic model fit was poor and necessitated use of a linear model to interpolate a value of PC_150._ Removing this subject from analyses did not materially change the results obtained. Additionally, seven preCC mice were almost completely unresponsive to methacholine, precluding identification of a PC_150_ value; these mice were therefore removed from the analyses, resulting in 146 preCC mice with complete phenotype data. These data are provided in File S2.

PC_150_ values for each mouse were calculated for before and after HDM treatment, and we refer to these as “baseline PC_150_” and “final PC_150_”, respectively, and we calculated the change in PC_150_ due to allergen (“delta PC_150_”) as baseline PC_150_–final PC_150_. For most mice, delta PC_150_ is positive because of heightened responsiveness to methacholine due to HDM treatment. One mouse showed a large, negative value of delta PC_150_ but inspection of the original Penh data for this mouse did not reveal any obvious abnormalities in the data, so we kept the mouse in the analysis. All PC_150_ values for preCC mice and CC founders are given in File S2. Box Cox transformation analyses indicated that log transformations of baseline and final PC_150_ were suitable for subsequent analyses (we used natural log (ln) transformations), while delta PC_150_ was left untransformed.

### Lung resistance data

We conducted invasive measurements of total lung resistance in two inbred strains of contrasting haplotypes on chromosome 19, C57BL/6J and C57BL/6J-Chr19^A^/NaJ, using the FinePointe Resistance and Compliance instrument from Data Sciences International. After allergen sensitization and challenge as above, mice were anesthetized with urethane (2 g/kg), and then administered pancuronium bromide (0.8 mg/kg) to prevent voluntary breathing once connected to the instrument. After a 5 min period of acclimation, increasing doses of methacholine (0, 6.25, 12.5, and 25 mg/ml) were administered via a nebulizer for 10 sec followed by measurements of resistance every 2 sec for 3 min. Lung resistance (R_L_) values for every measurement cycle over the 3 min recording period are reported PC_150_ values were calculated for each mouse as described above after collapsing the resistance data to mean values of resistance per dose of methacholine.

### Genotyping and QTL mapping

Genotypes for all of the preCC mice used in these experiments have been previously reported (Rutledge *et al.* 2014). We genotyped each mouse at the University of North Carolina – Chapel Hill, using one of two Affymetrix SNP arrays (A or B) that were produced in development of the Mouse Diversity array (MDA) (Yang *et al.* 2009). After removing uninformative and poorly performing SNPs, these arrays contained 181,752 (A-array) and 180,976 (B-array) SNP assays, and the set of SNPs on each array did not overlap. Most mice (83%) were genotyped on the B-array and the remaining were genotyped on the A-array. These training arrays were annotated to NCBI Build 36 of the mouse genome, but we mapped QTL boundaries to Build 37 positions to integrate with other resources. We report NCBI Build 37 positions in our results. We estimated the most probable ancestor for each SNP in each mouse using the GAIN algorithm (Liu *et al.* 2010), and reconstructed founder haplotypes based on these results. We then merged the nonoverlapping SNP datasets from arrays A and B by imputing unobserved genotypes based on inferred founder haplotype, resulting in ∼360,000 SNP markers in total. For QTL mapping, we used HAPPY (Mott *et al.* 2000) to infer ancestry matrices for an additive genetic model. For efficiency, we then averaged the matrices across SNPs between which GAIN inferred no recombination in the population, and this reduced the mapping dataset to 27,059 intervals with an average spacing of 95 kilobases. We used BAGPIPE (Valdar *et al.* 2006) to fit a regression model for log-transformed PC_150_ and report LOD scores. Significance thresholds were determined by permutation (*n* = 10,000). We used the 1.5 LOD drop method to approximate confidence intervals for QTL (Dupuis and Siegmund 1999). The percent of phenotypic variation explained by each QTL was estimated by regression of phenotypes on haplotype probabilities at the peak locus.

### Data availability

Supporting information is provided in two files. File S1 (FileS1_phenodata_penh.xls) contains Penh data for each mouse used in the study. File S2 (FileS2_phenodata2_pc150.xls) contains PC_150_ values calculated for each mouse in the study. Genotype data are publically available at http://www.genetics.org/content/198/2/735.supplemental.

## Results

**Figure 2:**
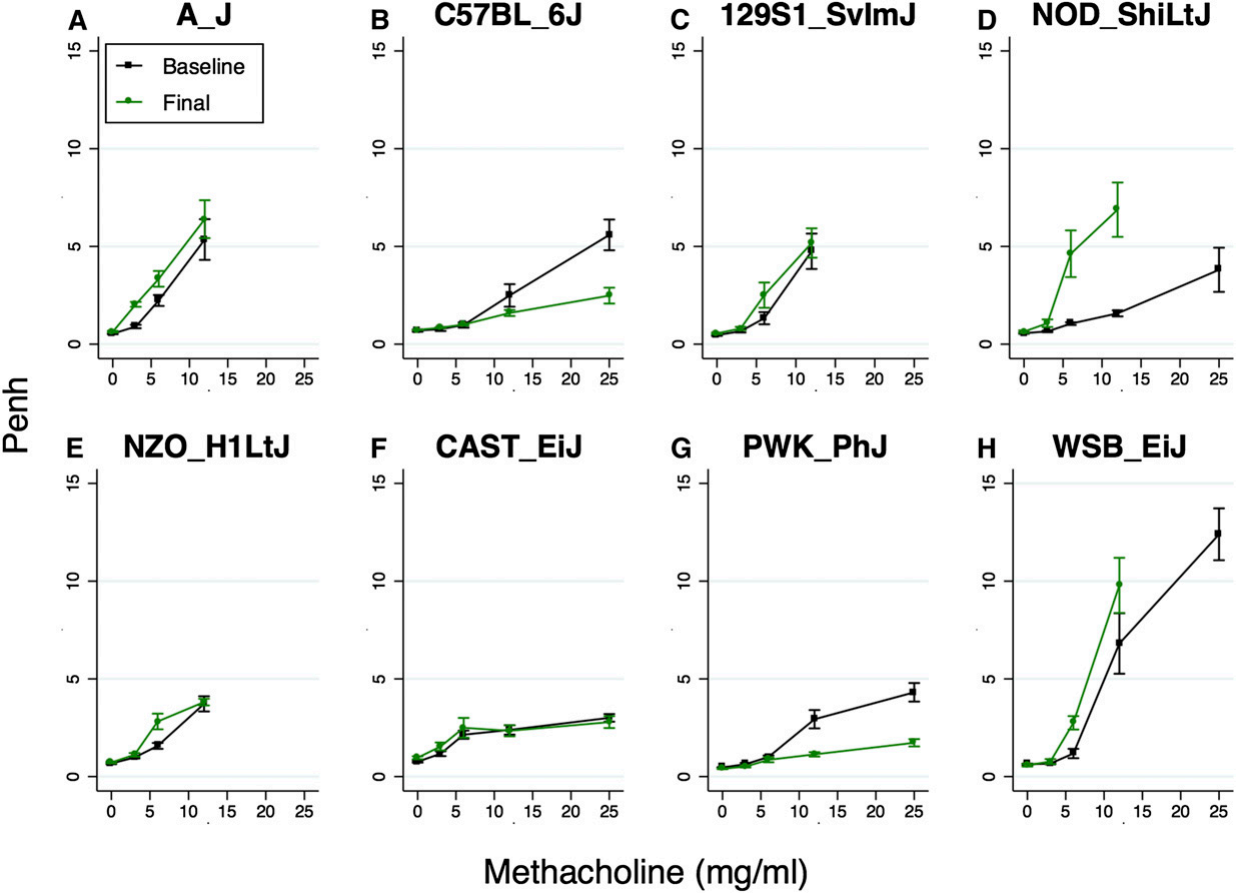
Baseline (black) and Final (green) Penh among CC founder strains. Data from (A) A/J, (B) C57BL/6J, (C) 129S1/SvImJ, (D) NOD/ShiLtJ, (E) NZO/H1LtJ, (F) CAST/EiJ, (G) PWK/PhJ, and (H) WSB/EiJ strains, with n = 5-9 male mice per strain. Mean and standard errors are shown. Note that mice from the A/J, 129S1/SvImJ, and NZO/H1LtJ strains exhibited initial signs of difficulty breathing at 12.5 mg/ml methacholine, so we did not administer the next dose of methacholine (25 mg/ml) to those mice and thus there are no Penh data for those strains at the highest dose.

The longitudinal study design used is shown in Figure 1. Two baseline WBP measurements were made, followed by sensitization and challenge with the HDM allergen Der p 1 over the course of 25 d. At the study conclusion 3 d later, a final WBP measurement was made followed by lung lavage and lung tissue collection. Five to nine mice from each CC founder strain and 146 male preCC mice were phenotyped in this manner. Baseline and final Penh values for each CC founder strain are shown in Figure 2. As expected based on previous findings (Levitt and Mitzner 1988; Duguet *et al.* 2000), the A/J strain showed the largest increases in Penh at low doses of methacholine, though WSB/EiJ mice exhibited remarkably high Penh values at high doses of methacholine.

**Figure 3:**
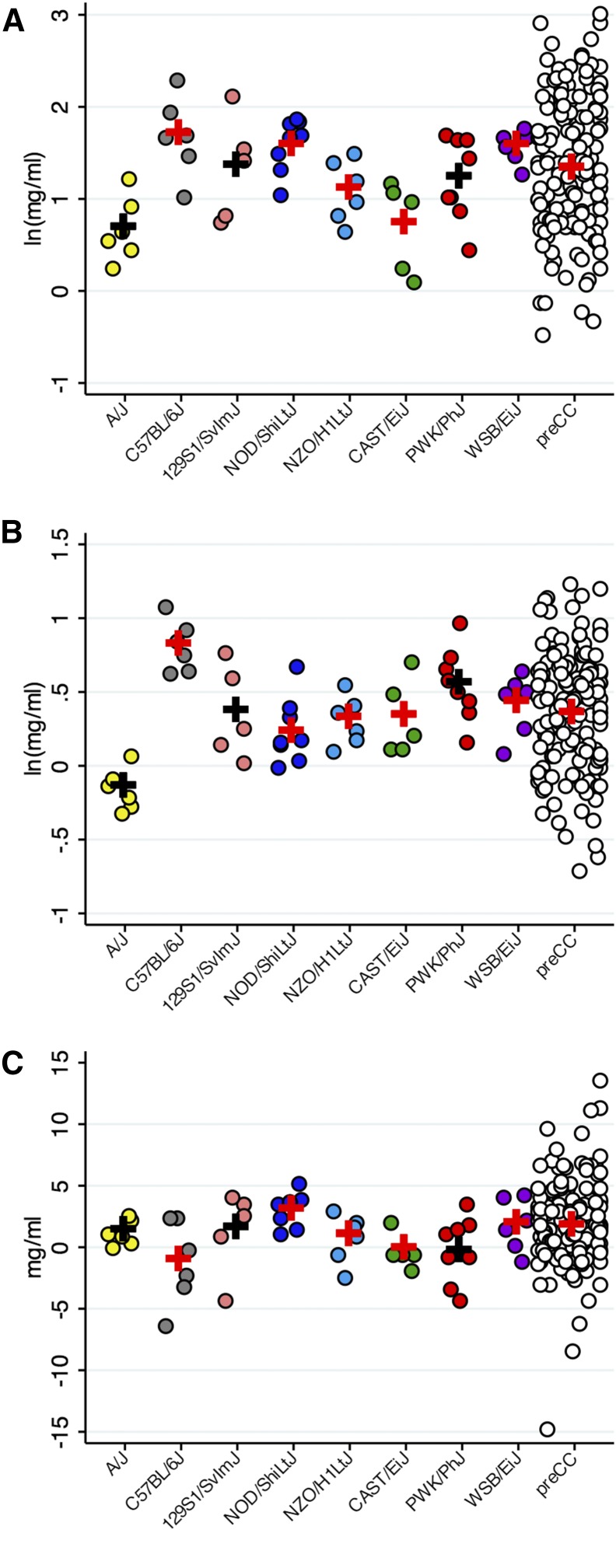
PC_150_ phenotype distributions among CC founder strains and preCC mice. Log-transformed values are shown for baseline PC_150_ (A), final PC_150_ (B), delta PC_150_ (C). *n* = 146 preCC mice. ANOVA *P*-values for differences among CC founder strains are 8.9 × 10^−5^, 1.19 × 10^−5^, and 0.33 for baseline PC_150_, final PC_150,_ and delta PC_150_ respectively. For delta PC_150,_ the population mean among preCC mice is −0.7 mg/ml.

We employed the same protocol with preCC mice, and we note that repeated measures of baseline Penh values separated by 1 wk were highly reproducible on a mouse-by-mouse basis, as shown in Figure S1. As with other AAD phenotypes (Kelada *et al.* 2014; Rutledge *et al.* 2014), there was considerable variation in Penh values among preCC mice. For both preCC mice and CC founder strains, we modeled the data as percent of Penh at baseline using a quadratic function to estimate the dose of methacholine required to produce a 50% increase in Penh, which we refer to as PC_150_, thus condensing the dose–response data into a single phenotype value. Example methacholine dose–response curves and quadratic model fitting for preCC mice are shown in Figure S1 and Figure S2. Overall, there were significant differences in baseline PC_150_ among CC founder strains (Figure 3A, ANOVA test *P*-value = 2.4 × 10^−4^). Importantly, the range of baseline PC_150_ values among preCC mice, which spanned two orders of magnitude (0.6 mg/ml to 20.3 mg/ml), was considerably greater than what we observed for CC founder strains (range = 1.1–10.8 mg/ml).

WBP measurements conducted after allergen sensitization and challenge likewise revealed a wide range of responses among CC founder strains and preCC mice (Figure 3B). Among CC founder strains, all strains except CAST/EiJ exhibited significant changes in PC_150_ due to the induction of AAD (Table 1 and Figure 2F). The global difference in final PC_150_ among founder strains was also more statistically significant compared to baseline PC_150_ (ANOVA *P*-value 6.2 × 10^−7^), and we note in particular that the differences between the two strains with the most extreme baseline PC150 values, A/J and C57BL/6J, were heightened due to AAD (*i.e.*, differences in mean final PC_150_ were greater than differences in baseline PC_150_). As with baseline PC_150_, the range of variation in final PC_150_ among preCC mice was greater than among CC founders (Figure 3B). Overall, allergen sensitization and challenge produced a significant change in PC_150_ at the population level among preCC mice (1.7 mg/ml change, *P* < 1x10^−4^).

**Table 1:** Baseline, final and delta PC_150_ values for CC founder strains and preCC mice

	PC_150_ (SE) in mg/ml			Comparison of Baseline *vs.* Final		
Strain	Baseline	Final	Delta	*t*-statistic	d.f.	*P*-value
A/J	2.09 (0.32)	0.75 (0.11)	1.35 (0.39)	4.59	5	5.9 × 10^−3^
C57BL/6J	5.98 (1.13)	7.04 (1.10)	−1.06 (1.49)	4.72	5	5.2 × 10^−3^
129S1/SvImJ	4.44 (1.24)	2.97 (1.03)	1.47 (1.50)	3.46	4	3.0 × 10^−2^
NOD/ShiLtJ	5.05 (0.49)	1.96 (0.45)	3.09 (0.45)	13.62	7	<1 × 10^−4^
NZO/H1LtJ	3.13 (0.39)	2.25 (0.42)	0.87 (0.69)	4.40	5	7.0 × 10^−4^
CAST/EiJ	2.24 (0.42)	2.48 (0.67)	−0.24 (0.65)	1.96	4	0.12
PWK/PhJ	3.70 (0.56)	3.96 (0.71)	−0.26 (1.05)	3.26	7	1.4 × 10^−4^
WSB/EiJ	4.94 (0.30)	2.97 (0.54)	1.97 (0.78)	8.05	5	5.0 × 10^−4^
preCC	4.87 (0.30)	3.19 (0.24)	1.67 (0.27)	21.19	150	<1 × 10^−4^

d.f., degrees of freedom.

For each mouse, we then calculated the change in PC_150_ (delta PC_150_) as baseline PC_150_–final PC_150_. For most preCC mice, delta PC_150_ is positive due to the effect of allergic inflammation on methacholine responsiveness, but some preCC mice exhibited decreases in PC_150_ after allergen sensitization and challenge (*e.g.*, Figure S3R), so delta PC_150_ is negative. Obtaining negative values of delta PC_150_ was not surprising given that two CC founder strains, C57BL/6J and PWK/PhJ, both exhibited decreased responses to higher doses of methacholine due to allergen sensitization and challenge (Table 1 and Figure 2, B and G) and we have previously documented decreased airway resistance due to allergen in C57BL/6J mice (Kelada *et al.* 2011). Differences in delta PC_150_ among CC founder strains were significant (ANOVA *P*-value = 0.04), but were not as robust as differences for baseline or final PC_150_. Notably, NOD/ShiLtJ mice exhibited the largest change in PC_150_ due to allergen with a 3.1 mg/ml difference in PC_150_, which equates to a 61% change.

Prior to QTL mapping, we examined other biological correlates of final PC_150_ and delta PC_150_. Specifically, we calculated pairwise correlations between final PC_150_ and delta PC_150_ with metrics of airway inflammation (Kelada *et al.* 2014; Rutledge *et al.* 2014) as well as baseline PC_150_ (Table 2). By far the strongest predictor of final PC_150_ was baseline PC_150_ (see also Figure S4). Final PC_150_ was also significantly correlated with eosinophil and lymphocyte counts, as well as concentrations of cytokines in lung lavage fluid, most prominently IL-5 and IL-10, and to a lesser extent IL-4 and IL-13. Surprisingly, delta PC_150_ was not correlated with metrics of inflammation; however delta PC_150_ was strongly correlated with baseline PC_150_.

**Table 2:** Correlations between final and delta PC_150_ values with markers of inflammation*^a^*

	Final PC_150_	delta PC_150_
Baseline PC_150_	0.58*^b^*	−0.61*^b^*
Final PC_150_	1	0.16*^g^*
Neutrophils	0.10	−0.04
Eosinophils	0.30*^e^*	0.03
Macrophages	0.10	−0.03
Lymphocytes	0.34*^d^*	0.04
IL-4	0.23*^f^*	0.00
IL-5	0.37*^c^*	−0.08
IL-6	0.26*^f^*	−0.05
IL-10	0.41*^b^*	−0.03
IL-13	0.26*^f^*	−0.01
MCP1	0.29*^e^*	−0.15
CCL11	0.15	0.06
CXCL1	0.15	−0.10
CXCL10	0.29*^e^*	0.02
TIMP1	0.26*^f^*	−0.02
IgE	0.14	0.00
IgG1	0.05	−0.07

a Spearman rho values for correlations with cell counts and cytokines (also measured 72 hr after allergen challenge) are shown.

b *P* < 1 × 10^−6^.

c *P* < 1 × 10^−5^.

d *P* < 1 × 10^−4^.

e *P* < 1 × 10^−3^.

f *P* < 1 × 10^−2^.

g *P* < 5 × 10^−2^.

**Figure 4:**
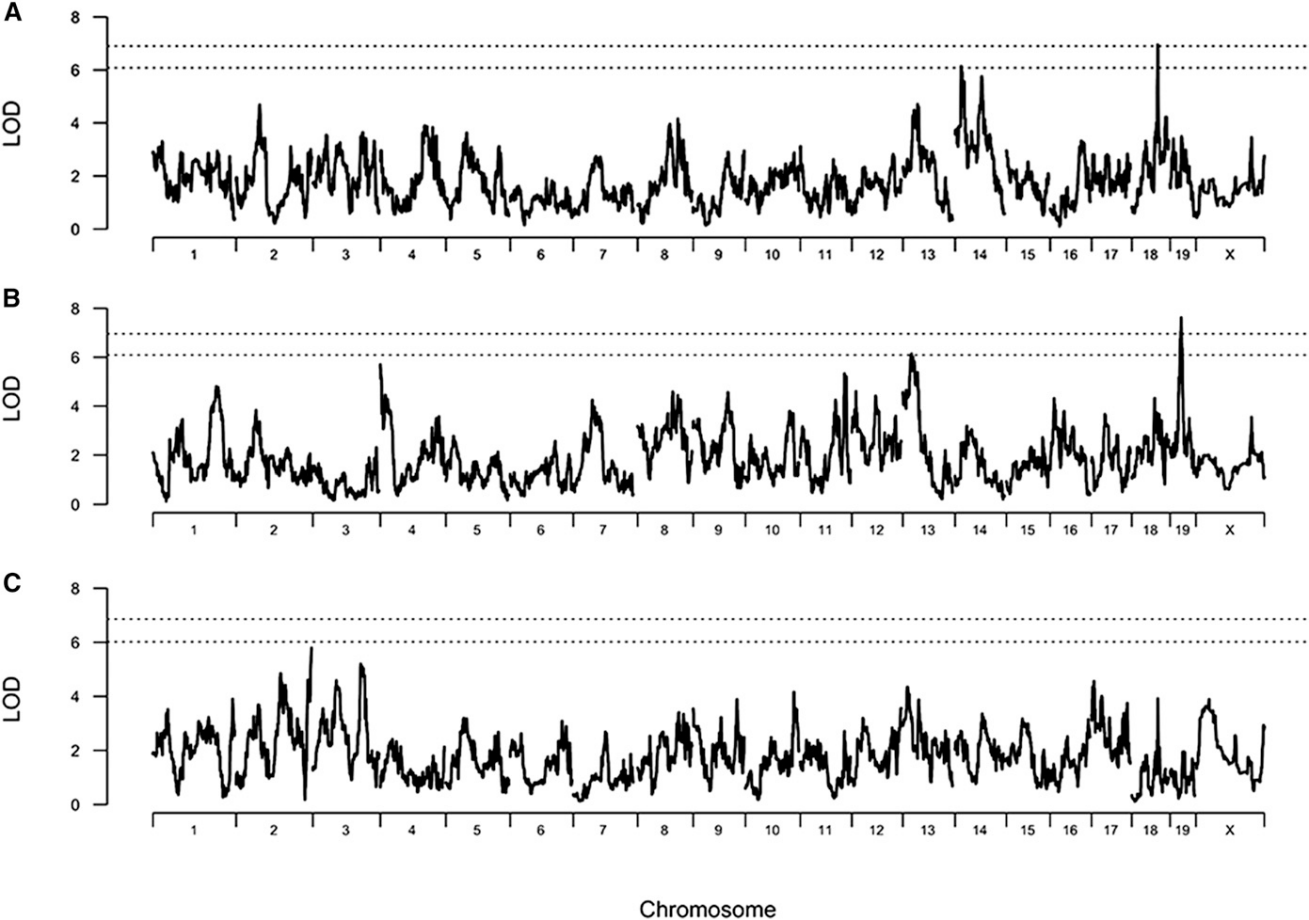
Genome scans for baseline (A), final (B) and delta (C) PC_150_ phenotypes. Dashed horizontal lines indicate 95% and 80% significance thresholds as determined by permutations.

We then performed QTL mapping using log (ln)-transformed values of baseline PC_150_ and final PC_150_, and untransformed values of delta PC_150_. Genome scans are shown in Figure 4 and a summary of QTL detected is presented in Table 3. We identified one significant and one suggestive QTL for baseline PC_150_ on chromosomes (Chr) 18 and 14, respectively. The allele effects for the Chr 18 QTL are shown in Figure S5. For final PC_150_, we identified one significant and one suggestive QTL on Chr 19 and 13, respectively; but we did not detect QTL for delta PC_150_. Chromosome region plots of the two significant QTL on Chrs 18 (baseline PC_150_) and 19 (final PC_150_) are shown in Figure S6 and Figure S7. These two QTL accounted for 20% and 21% of phenotypic variance, respectively.

**Table 3:** QTL for methacholine responsiveness before and after allergen

Phenotype	Threshold	chr	LOD	Peak*^a^*	95% CI Start*^a^*	95% CI End*^a^*
PC_150_ baseline	0.2	14	6.14	22.960	21.810	28.728
PC_150_ baseline	0.05	18	6.95	64.761	63.670	65.295
PC_150_ final	0.2	13	6.14	23.826	18.322	39.773
PC_150_ final	0.05	19	7.62	29.639	27.566	31.134

CI, confidence interval.

a Positions are in megabases.

The fact that QTL for baseline and final PC_150_ differed indicated that final PC_150_ is a distinct phenotype from baseline PC_150_ despite the fact that two phenotypes are strongly correlated. Additionally, the strong correlations between final PC_150_ and concentrations of interleukins 5 and 10 in lung lavage fluid (Table 2) suggest this QTL is associated with AAD. We focused our subsequent genetic analyses on the QTL for final PC_150_ since our primary interest is in the effect of allergen on airway responsiveness to methacholine.

**Figure 5:**
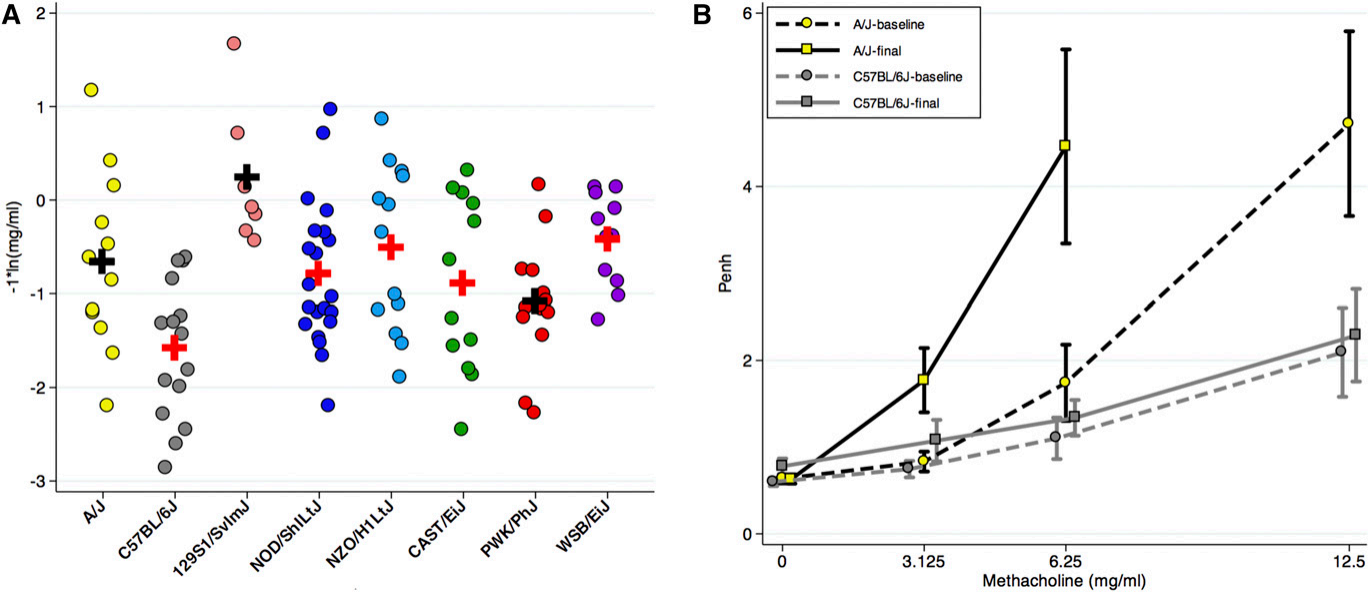
Allele effects for Chr 19 final PC_150_ QTL. (A) Final PC_150_ values *vs.* most probable founder haplotype for mice. Final PC_150_ values have been inverted (*−1) to facilitate interpretation of the data in part B. Only data for homozygotes are shown (*n* = 105). (B) Baseline and Final Penh by founder haplotype for mice with A/J (*n* = 12) or C57BL/6J (*n* = 16) founder haplotypes. Penh data acquired at higher methacholine doses are not shown because six mice with the A/J haplotype exhibited early signs of respiratory difficulty starting at 12.5 mg/ml, necessitating removal from the WBP chamber. X-axis values (for methacholine) have been slightly shifted to facilitate visualization of the data. An additional plot including mice with 129S1/SvImJ founder haplotype is shown in Figure S8.

We examined the allele effects for the Chr 19 QTL (Figure 5). Final PC_150_ differed among preCC mice of the different founder haplotypes (Figure 5A) with C57BL/6J haplotypes associated with largest final PC_150_ values. While not the largest difference between haplotypes, the contrast between C57BL/6J *vs.* A/J haplotypes was significant and noteworthy because it is consistent with the overall difference between the A/J and C57BL/6J founder strains (see Figure 2), suggesting this locus may explain a portion of the overall phenotypic variance between these two commonly used inbred strains.

To further examine this, we plotted raw baseline and final Penh values (*vs.* methacholine) as a function of most probable founder haplotype for preCC mice homozygous for A/J and C57BL/6J haplotypes at this locus (Figure 5B). After allergen sensitization and challenge, preCC mice with A/J founder haplotypes exhibited higher Penh values at low doses of methacholine, and at higher doses of methacholine several mice with this haplotype lack Penh data because they were pulled from the WBP chambers to prevent respiratory difficulty (note that this was done without knowledge of genotype). Thus both phenotype metrics, Penh values and PC_150_, indicate that the A/J haplotype at this locus confers higher responses to methacholine after allergen sensitization and challenge. A similar plot containing data from mice with the A/J, C57BL/6J, or 129S1/SvImJ haplotypes is provided in Figure S8.

**Figure 6:**
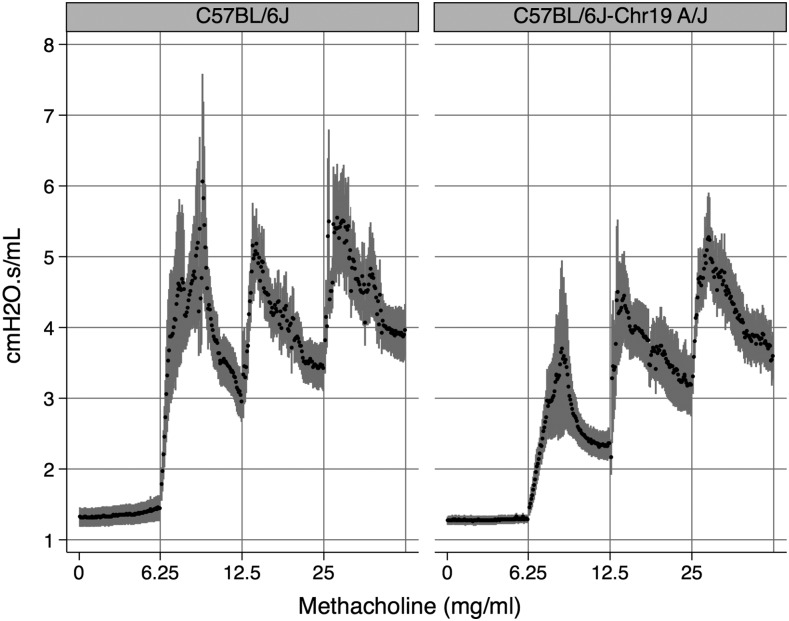
Lung resistance after HDM sensitization and challenge in C57BL/6J and C57BL/6J^Chr19|A/J|Na/J^ mice. Vertical gray lines represent points at which methacholine was administered, followed by 3 min of measurements. Black dots and gray bars represent mean and standard errors, respectively. Thirteen male C57BL/6J mice and nine C57BL/6J^Chr19 A/J Na/J^ mice were phenotyped.

To test whether the Chr 19 A/J haplotype confers increased resistance to airflow *per se*, we conducted invasive measurements of total lung resistance using male mice from two strains with contrasting Chr 19 haplotypes, C57BL/6J and C57BL/6J-Chr19^A^/NaJ, the latter coming from an established panel of consomic strains (Nadeau *et al.* 2000). We sensitized and challenged mice from both strains with house dust mite allergen and evaluated total lung resistance in response to methacholine challenge. As shown in Figure 6, resistance values in response to methacholine were not demonstrably different between the two strains. After collapsing the data to mean values of resistance per dose of methacholine (Figure S9), we calculated the values of PC_150_ for each strain and found that the difference between the two strains was not significant [0.58 ± 0.51 *vs.* 1.65 ± 0.57 (mean ± SE) for C57BL/6J and C57BL/6J-Chr19^A^/NaJ, respectively, *P*-value = 0.18]. These data argue against an appreciable role for the final PC_150_ QTL on lung resistance.

## Discussion

We employed a longitudinal study design in which partially inbred preCC mice and CC founder strains were phenotyped before and after the induction of AAD using WBP so that we could distinguish between high responses to methacholine due to strain alone *vs.* strain and allergen. Our results using CC founder strains indicate that the A/J strain is highly responsive to methacholine at baseline, corroborating the results of several previous studies (De Sanctis *et al.* 1995; Ackerman *et al.* 2005; Leme *et al.* 2010; Berndt *et al.* 2011; Kanagaratham *et al.* 2014), and even more so after allergen sensitization and challenge. Perhaps the most interesting finding from studies with CC founders was that the NOD/ShiLtJ strain showed the largest change in methacholine response as a function of allergen sensitization and challenge. Few studies of AAD have used this strain (Araujo *et al.* 2004; Aumeunier *et al.* 2010), but our results suggest much could be gained by using this strain to identify novel, potentially epistatic (Lin *et al.* 2013), QTL for AAD.

Studies with the preCC mice led to four primary conclusions. First, as with most traits studied to date (Aylor *et al.* 2011; Kelada *et al.* 2012, 2014; Rutledge *et al.* 2014), there is a wider range of methacholine responses in the preCC population than in the CC founder strains, both at baseline and after allergen challenge. This result indicates that the CC breeding design yielded preCC mice with combinations of alleles that collectively yield more extreme phenotypes. This was particularly apparent in the case of preCC mice that responded to low doses of methacholine with high Penh values; in some cases mice had been removed from WBP chambers to prevent respiratory distress at doses as low as 6.25 mg/ml. Second, we found that there was a strong, negative correlation between the change in PC_150_ values due to allergen (delta PC_150_) and baseline PC_150_ among preCC mice. The mice that exhibited large changes in PC_150_ were ones that initially were not very responsive to methacholine, which is perhaps intuitive. Third, we found that postallergen PC_150_ was significantly correlated with concentrations of cytokines and chemokines known to be related to AAD, such as classic T_h_2 interleukins 4, 5, and 13. Fourth, we found that distinct loci regulate pre- and postallergen responses to methacholine, consistent with the hypothesis that gene–environment interactions contribute to the allergen response. The QTL we identified on Chrs 18 and 19 have not been identified in previous studies in which WBP was used (Camateros *et al.* 2010; Berndt *et al.* 2011). The novelty of these loci is likely attributable to the genetic diversity present in the CC founder strains and the effective randomization of the founder genomes. The lack of QTL for delta PC_150_ was not surprising given that the differences among CC founder strains were not as robust as we observed for baseline and final PC_150_. This may be due to the fact that the delta PC_150_ phenotype includes measurement error (or noise) from two serial WBP measurements, not just one.

Despite the fact that postallergen PC_150_ was significantly correlated with hallmark biomarkers of AAD (*e.g.*, eosinophils, lymphocytes, IL-5, and IL-10 in lung lavage fluid), the QTL we identified for postallergen PC_150_ on Chr 19 was not validated in subsequent experiments using invasive measurements of airway mechanics with two strains of contrasting haplotypes in the region of interest. There has been considerable debate in the literature about whether Penh is reflective of airway resistance. Indeed a direct comparison of results using WBP *vs.* invasive measurements of resistance in a large set of inbred strains at baseline did not show concordance between methods (Berndt *et al.* 2011). Likewise, our findings generated using a genetically diverse population of mice treated to induce AAD, suggest that while Penh is correlated with inflammation phenotypes, it is not predictive of airway resistance and therefore should not be used in these types of studies.

While the results from Chr 19 consomic mice argue against the locus on Chr 19 being a *bona fide* QTL for airway hyper-responsiveness, we cannot exclude the possibility that a second locus on Chr 19 had an effect on the observed phenotypes, obscuring the effect of the locus we sought to validate. That is, it is possible that an A/J haplotype on proximal or distal Chr 19 dampens the effect of the QTL at 30 Mb. The use of the C57BL/6J-Chr19^A^/NaJ strain precludes an evaluation of the effect of the Chr19 locus dissociated from other regions on Chr 19. Use of currently available CC strains would not address this issue either, as by and large most CC strains with an A/J haplotype at 30 Mb on Chr 19 have A/J haplotypes for the vast majority of the chromosome.

Now that inbred CC lines are established, direct comparisons of airway resistance as a function of strain and allergen treatment can be conducted, offering a chance for a more robust investigation of the role of gene–allergen interactions in airway resistance. Additionally, results from these studies will offer an opportunity to compare the genetic architecture of arguably the most important aspect of allergic airway inflammation in mouse models and human disease.

## Supplementary Material

Supplemental Material
