# Supplementary material for: Plethysmography Phenotype QTL in Mice Before and After Allergen Sensitization and Challenge
Source: G3 (Bethesda). 2016 Jul 21;6(9):2857–65. doi: 10.1534/g3.116.032912 (PMC5015943; doi:10.1534/g3.116.032912)
Supplement: Supplemental Material [file supp_6_9_2857__index.html]

Plethysmography Phenotype QTL in Mice Before and After Allergen Sensitization and Challenge — Supplemental Material 

# Plethysmography Phenotype QTL in Mice Before and After Allergen Sensitization and Challenge

## Supplemental Material for Kelada, 2016

**Files in this Data Supplement:**

- Figure S1 - Example Penh data. (.pptx, 213 KB)
- Figure S2 - Observed data and quadratic fit of Penh expressed as percent of control (methacholine dose = 0). (.pptx, 253 KB)
- Figure S3 - Example preCC baseline and final Penh data demonstrating a range of responses to sensitization and challenge with house dust mite allergen. (.pptx, 170 KB)
- Figure S4 - Pairwise scatterplots of baseline, final and delta PC150. (.pptx, 173 KB)
- Figure S5 - Allele effects for Chr 18 baseline PC150 QTL. (.pptx, 101 KB)
- Figure S6 - Zoomed in QTL plot for Chr 18 baseline PC150 QTL. (.pptx, 103 KB)
- Figure S7 - Zoomed in QTL plot for Chr 19 final PC150 QTL. (.pptx, 107 KB)
- Figure S8 - Baseline and Final Penh by founder haplotype for mice with A/J, C57BL/6J, or 129S1/SvImJ founder haplotypes. (.pptx, 89 KB)
- Figure S9 - Mean lung resistance after HDM sensitization and challenge in C57BL/6J and C57BL/6JChr19 A/J Na/J mice. (.pptx, 104 KB)
- File S1 - Penh data for each mouse used in the study. (.xls, 94 KB)
- File S2 - PC150 values calculated for each mouse in the study. (.xls, 26 KB)
