# Supplementary material for: Plethysmography Phenotype QTL in Mice Before and After Allergen Sensitization and Challenge
Source: G3 (Bethesda). 2016 Jul 21;6(9):2857–65. doi: 10.1534/g3.116.032912 (PMC5015943; doi:10.1534/g3.116.032912)
Supplement: Supplemental Material [file supp_g3.116.032912_FigureS1.pptx]

## Slide 1
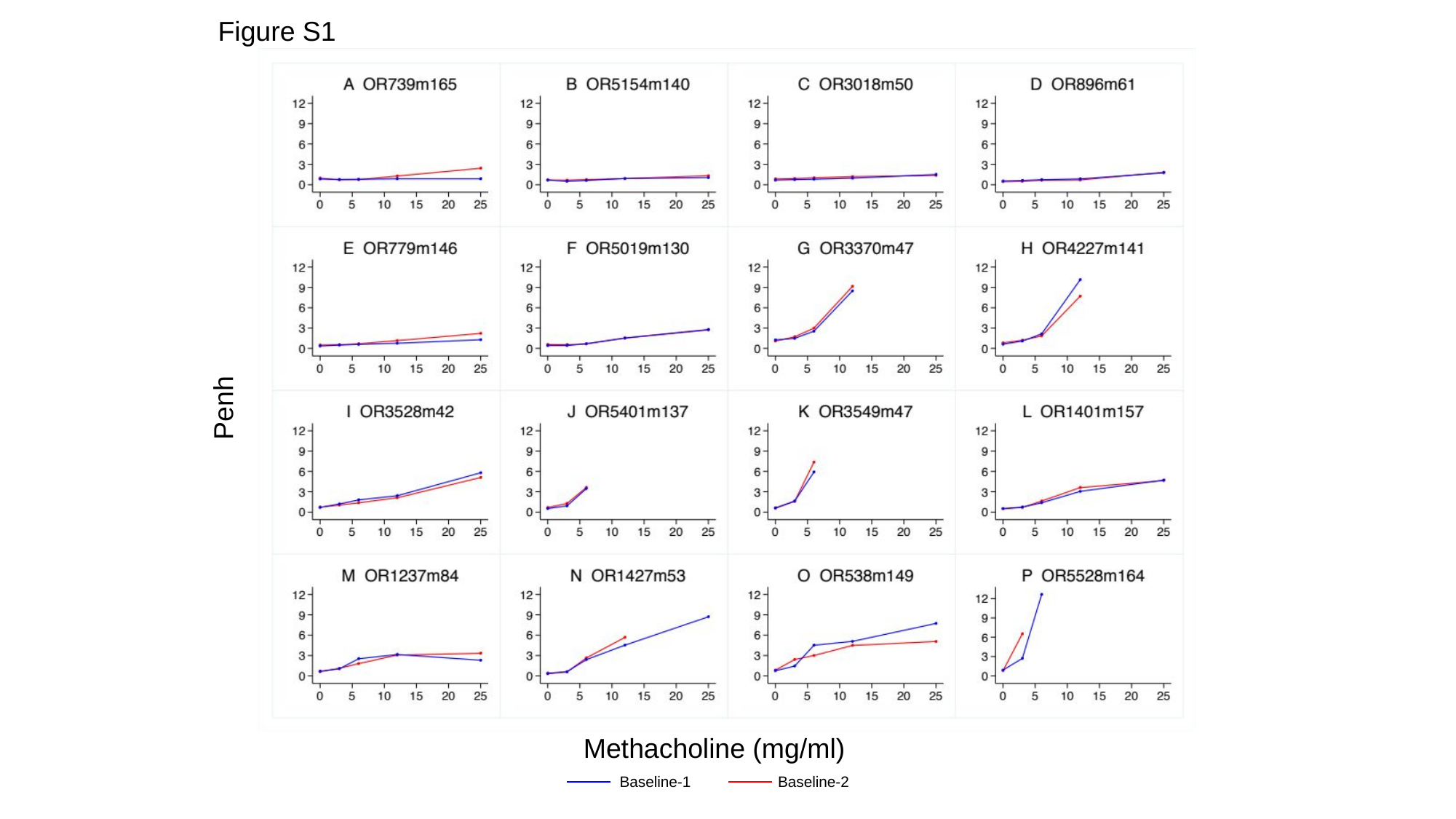

Figure S1
Penh
Methacholine (mg/ml)
Baseline-1
Baseline-2

## Slide 2
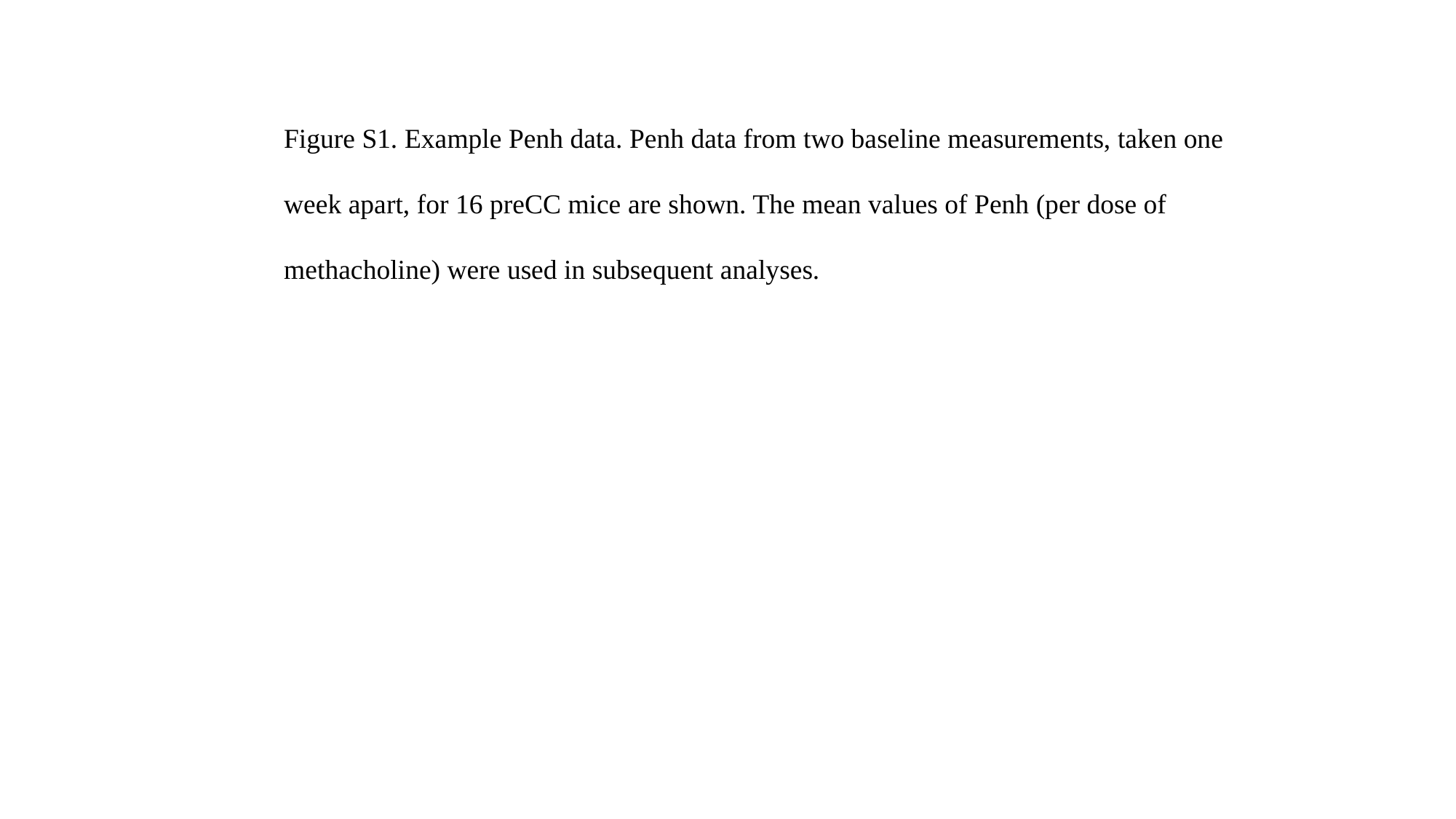

Figure S1. Example Penh data. Penh data from two baseline measurements, taken one week apart, for 16 preCC mice are shown. The mean values of Penh (per dose of methacholine) were used in subsequent analyses.
