# Supplementary material for: Plethysmography Phenotype QTL in Mice Before and After Allergen Sensitization and Challenge
Source: G3 (Bethesda). 2016 Jul 21;6(9):2857–65. doi: 10.1534/g3.116.032912 (PMC5015943; doi:10.1534/g3.116.032912)
Supplement: Supplemental Material [file supp_g3.116.032912_FigureS2.pptx]

## Slide 1
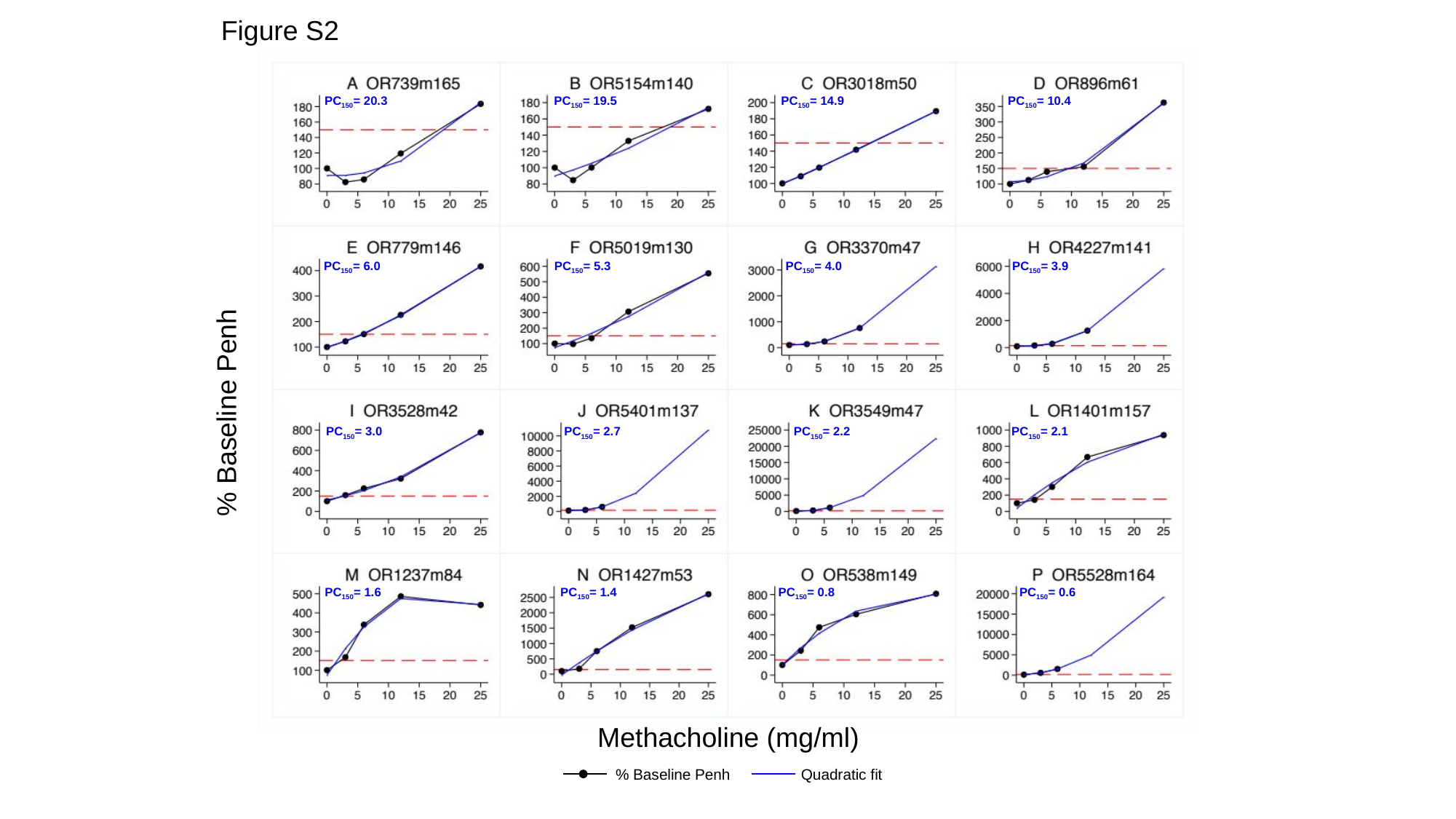

Figure S2
PC150= 20.3
PC150= 19.5
PC150= 14.9
PC150= 10.4
PC150= 6.0
PC150= 5.3
PC150= 4.0
PC150= 3.9
% Baseline Penh
PC150= 3.0
PC150= 2.7
PC150= 2.2
PC150= 2.1
PC150= 1.6
PC150= 1.4
PC150= 0.8
PC150= 0.6
Methacholine (mg/ml)
% Baseline Penh
Quadratic fit

## Slide 2
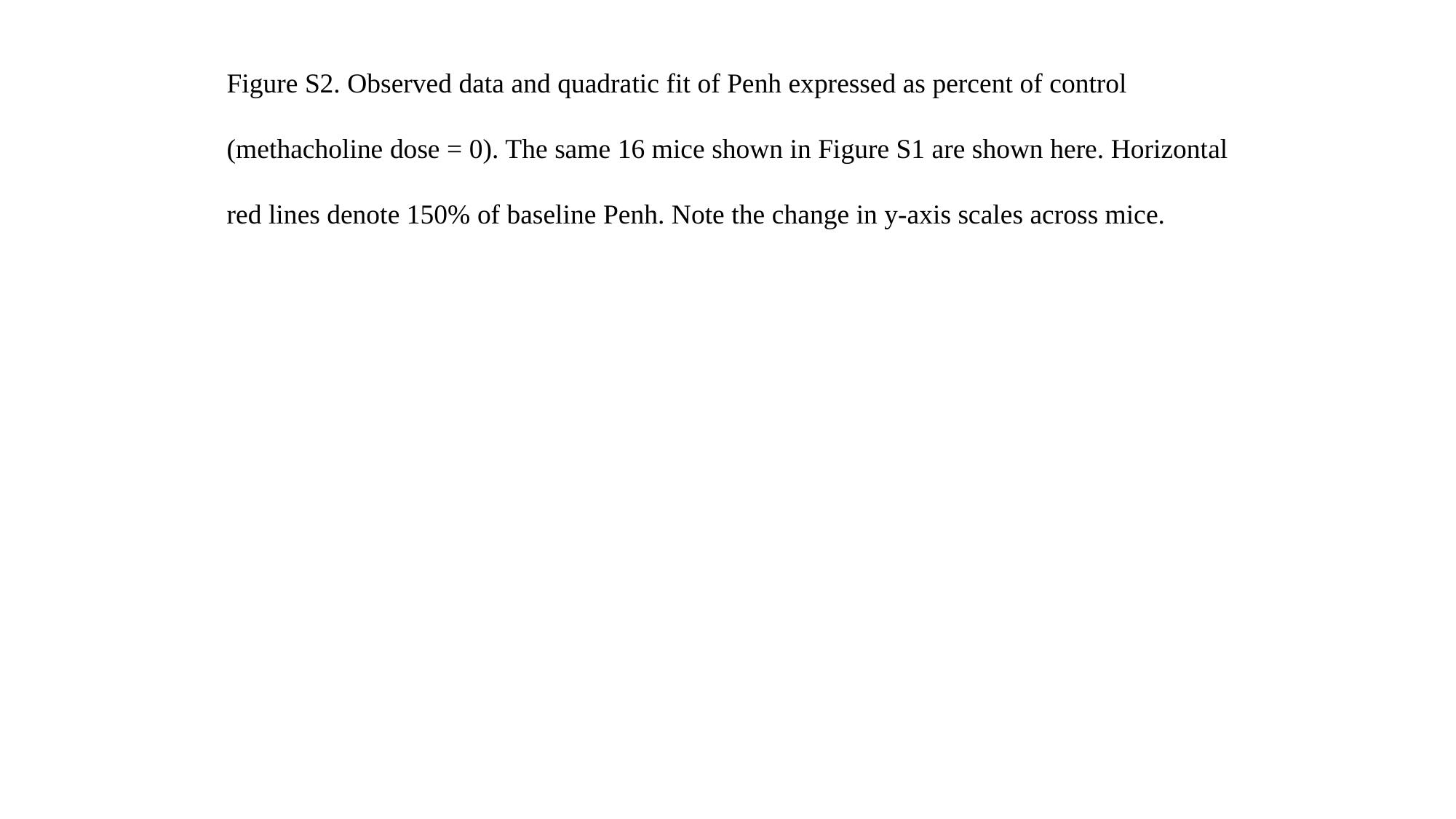

Figure S2. Observed data and quadratic fit of Penh expressed as percent of control (methacholine dose = 0). The same 16 mice shown in Figure S1 are shown here. Horizontal red lines denote 150% of baseline Penh. Note the change in y-axis scales across mice.
