# Supplementary material for: Plethysmography Phenotype QTL in Mice Before and After Allergen Sensitization and Challenge
Source: G3 (Bethesda). 2016 Jul 21;6(9):2857–65. doi: 10.1534/g3.116.032912 (PMC5015943; doi:10.1534/g3.116.032912)
Supplement: Supplemental Material [file supp_g3.116.032912_FigureS3.pptx]

## Slide 1
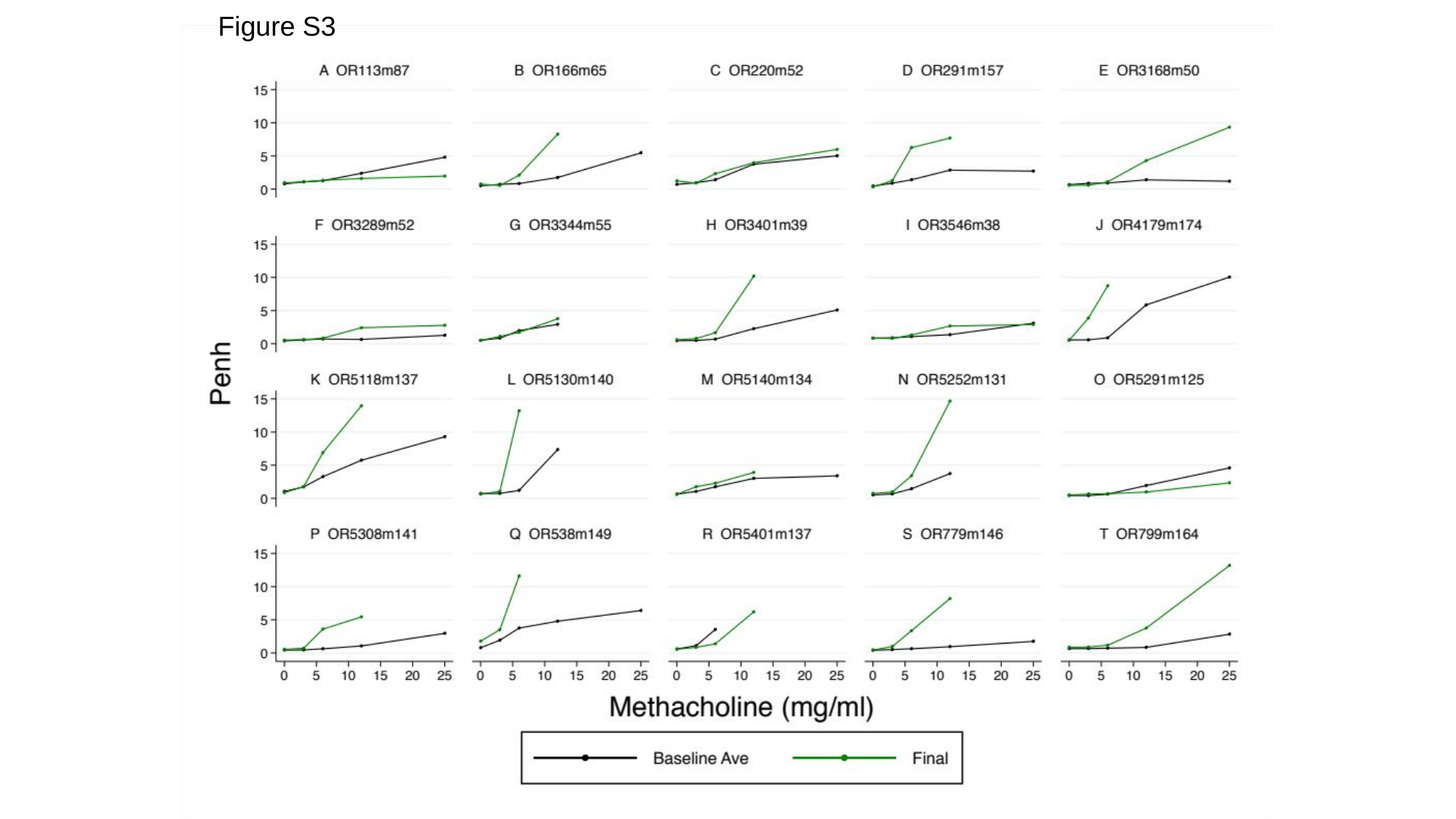

Figure S3

## Slide 2
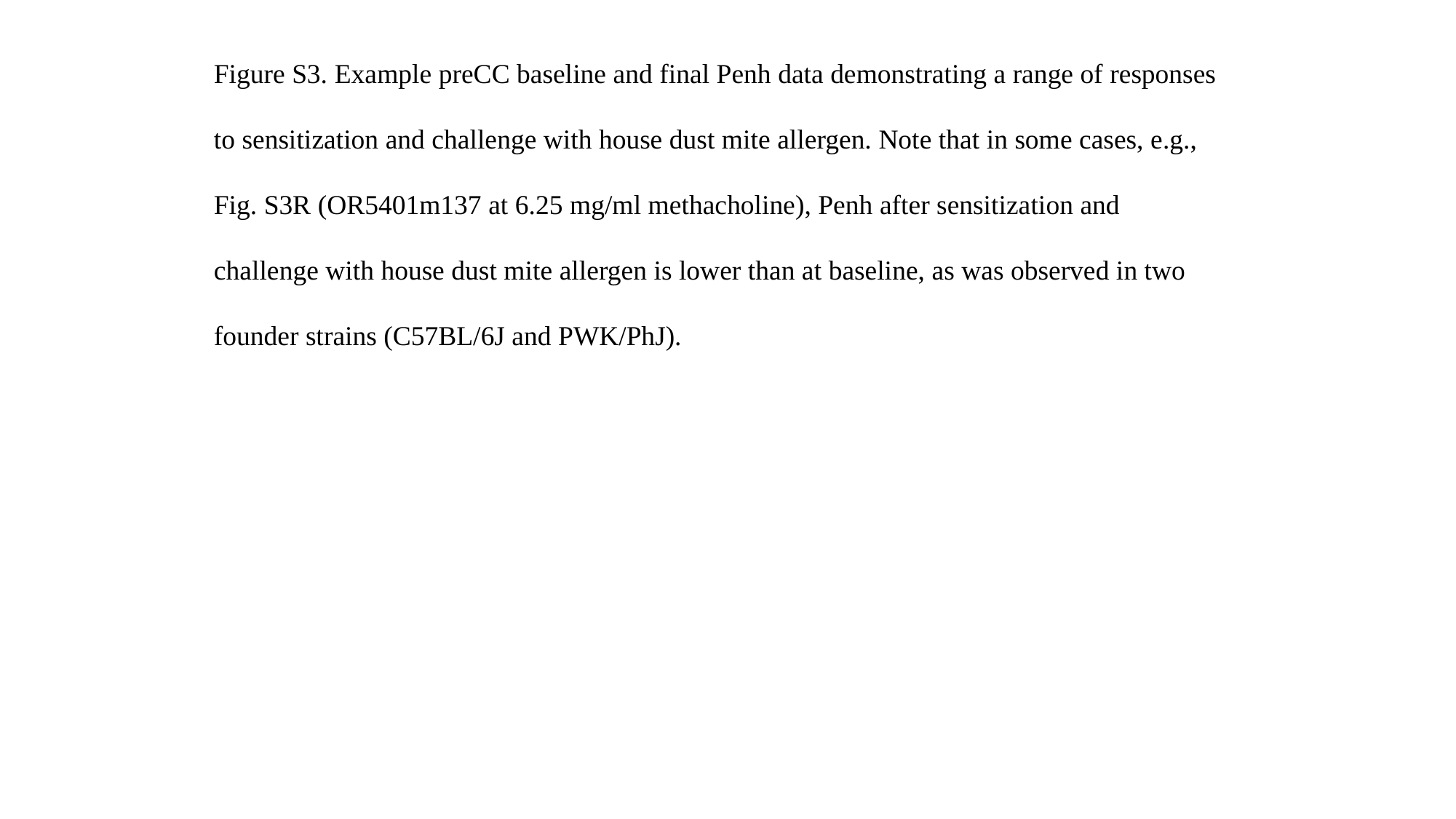

Figure S3. Example preCC baseline and final Penh data demonstrating a range of responses to sensitization and challenge with house dust mite allergen. Note that in some cases, e.g., Fig. S3R (OR5401m137 at 6.25 mg/ml methacholine), Penh after sensitization and challenge with house dust mite allergen is lower than at baseline, as was observed in two founder strains (C57BL/6J and PWK/PhJ).
