# Supplementary material for: Plethysmography Phenotype QTL in Mice Before and After Allergen Sensitization and Challenge
Source: G3 (Bethesda). 2016 Jul 21;6(9):2857–65. doi: 10.1534/g3.116.032912 (PMC5015943; doi:10.1534/g3.116.032912)
Supplement: Supplemental Material [file supp_g3.116.032912_FigureS4.pptx]

## Slide 1
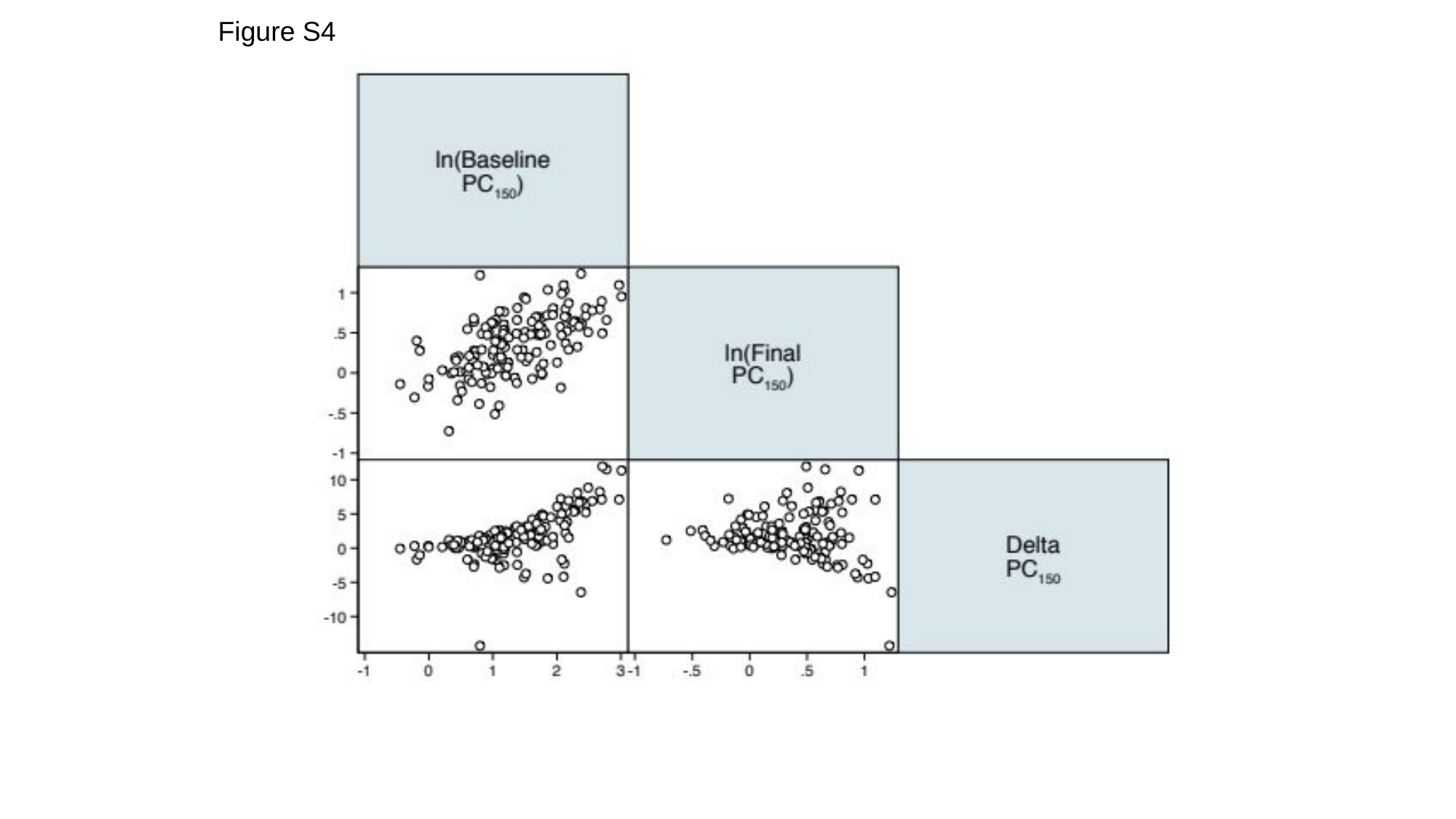

Figure S4

## Slide 2
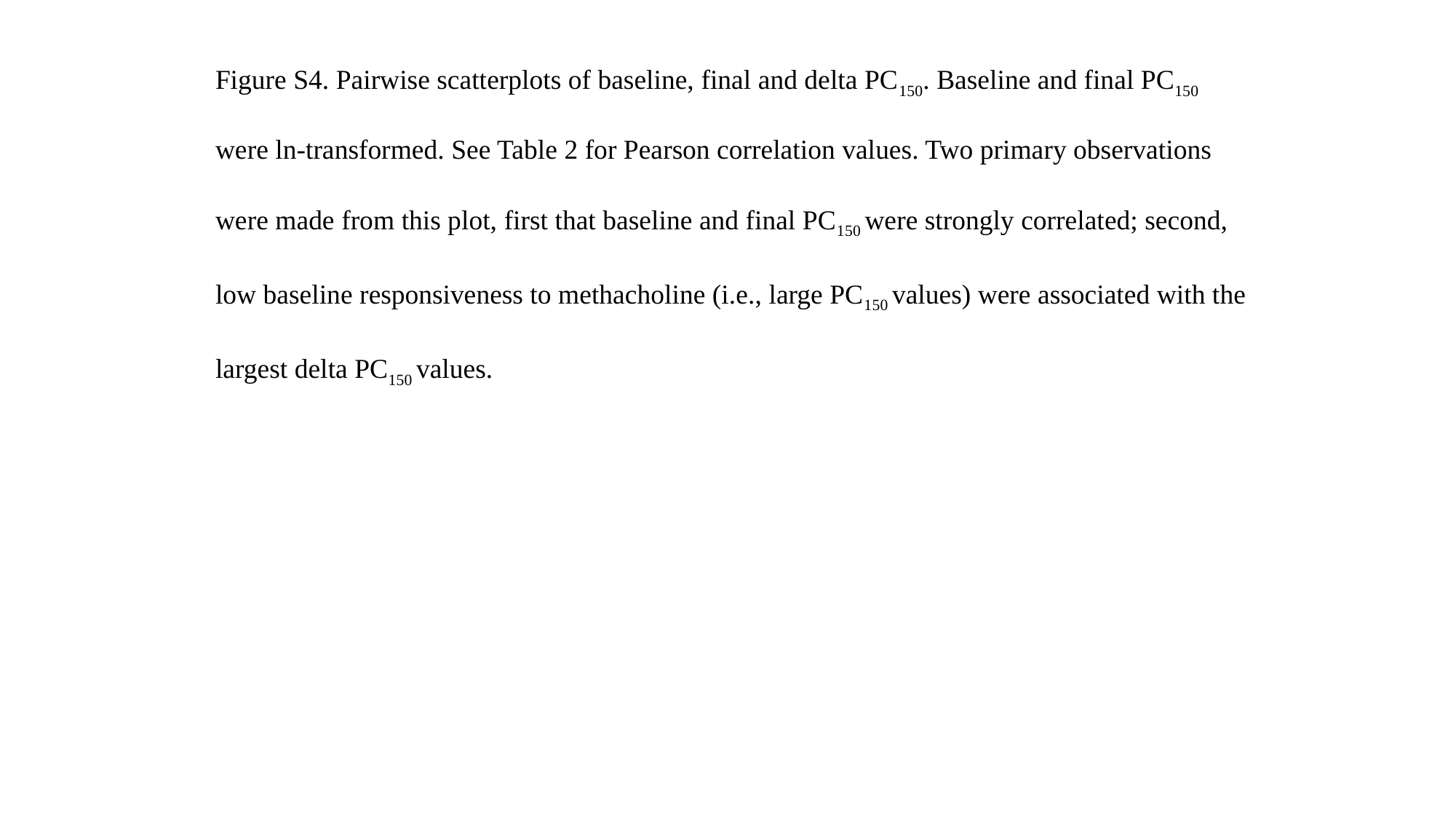

Figure S4. Pairwise scatterplots of baseline, final and delta PC150. Baseline and final PC150 were ln-transformed. See Table 2 for Pearson correlation values. Two primary observations were made from this plot, first that baseline and final PC150 were strongly correlated; second, low baseline responsiveness to methacholine (i.e., large PC150 values) were associated with the largest delta PC150 values.
