# Supplementary material for: Plethysmography Phenotype QTL in Mice Before and After Allergen Sensitization and Challenge
Source: G3 (Bethesda). 2016 Jul 21;6(9):2857–65. doi: 10.1534/g3.116.032912 (PMC5015943; doi:10.1534/g3.116.032912)
Supplement: Supplemental Material [file supp_g3.116.032912_FigureS5.pptx]

## Slide 1
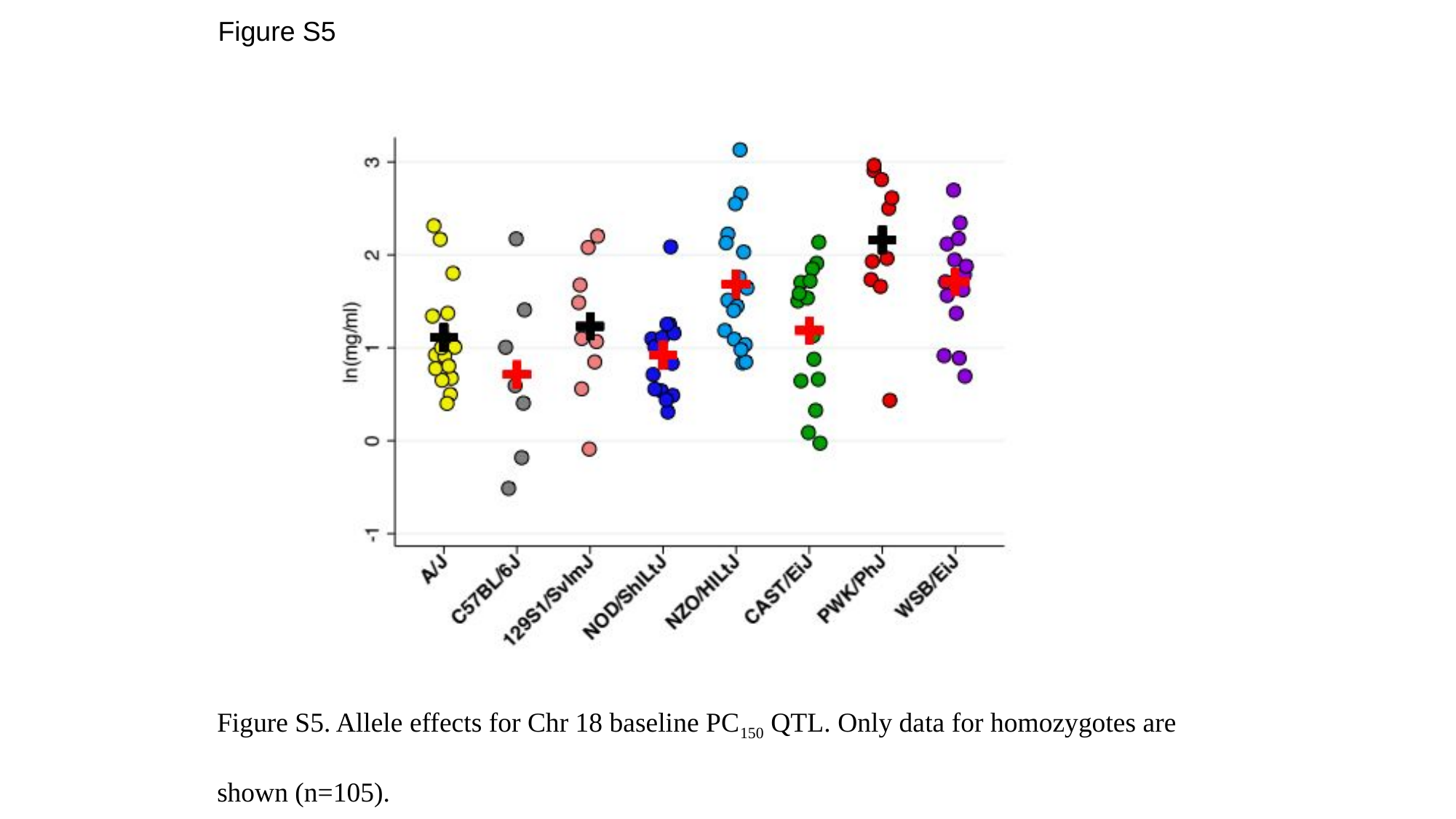

Figure S5
Figure S5. Allele effects for Chr 18 baseline PC150 QTL. Only data for homozygotes are shown (n=105).
