# Supplementary material for: Plethysmography Phenotype QTL in Mice Before and After Allergen Sensitization and Challenge
Source: G3 (Bethesda). 2016 Jul 21;6(9):2857–65. doi: 10.1534/g3.116.032912 (PMC5015943; doi:10.1534/g3.116.032912)
Supplement: Supplemental Material [file supp_g3.116.032912_FigureS6.pptx]

## Slide 1
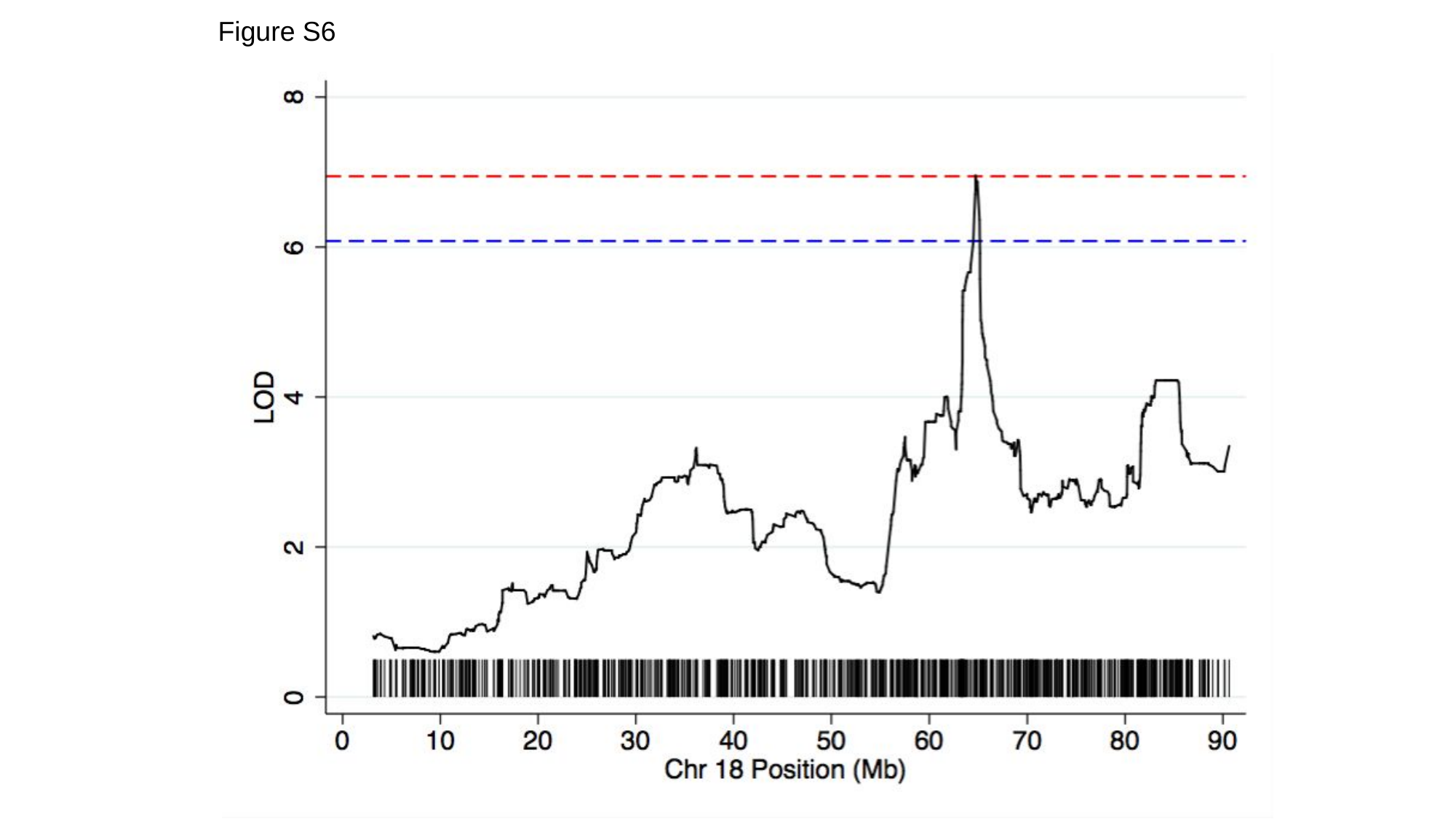

Figure S6

## Slide 2
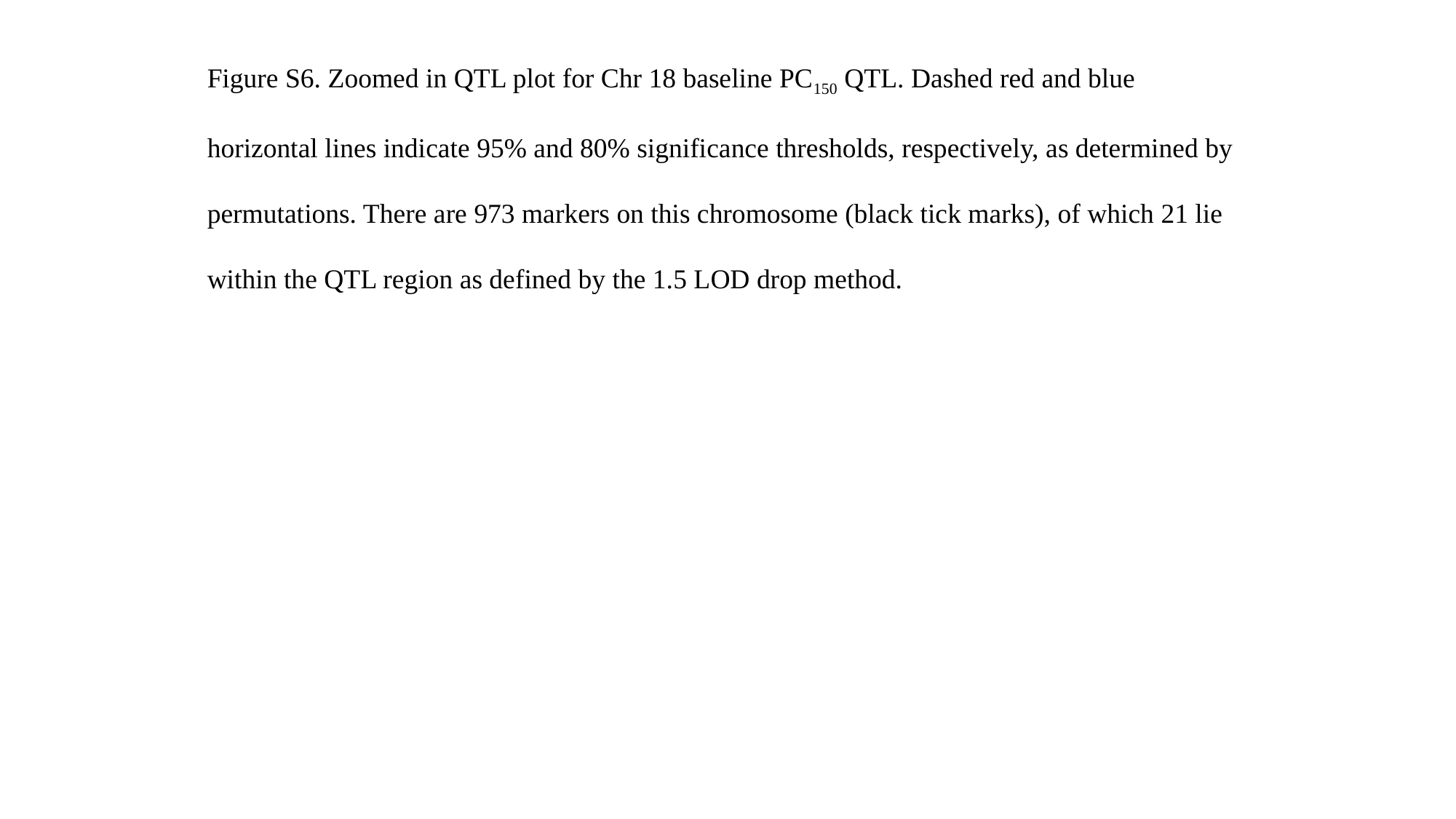

Figure S6. Zoomed in QTL plot for Chr 18 baseline PC150 QTL. Dashed red and blue horizontal lines indicate 95% and 80% significance thresholds, respectively, as determined by permutations. There are 973 markers on this chromosome (black tick marks), of which 21 lie within the QTL region as defined by the 1.5 LOD drop method.
