# Supplementary material for: Plethysmography Phenotype QTL in Mice Before and After Allergen Sensitization and Challenge
Source: G3 (Bethesda). 2016 Jul 21;6(9):2857–65. doi: 10.1534/g3.116.032912 (PMC5015943; doi:10.1534/g3.116.032912)
Supplement: Supplemental Material [file supp_g3.116.032912_FigureS8.pptx]

## Slide 1
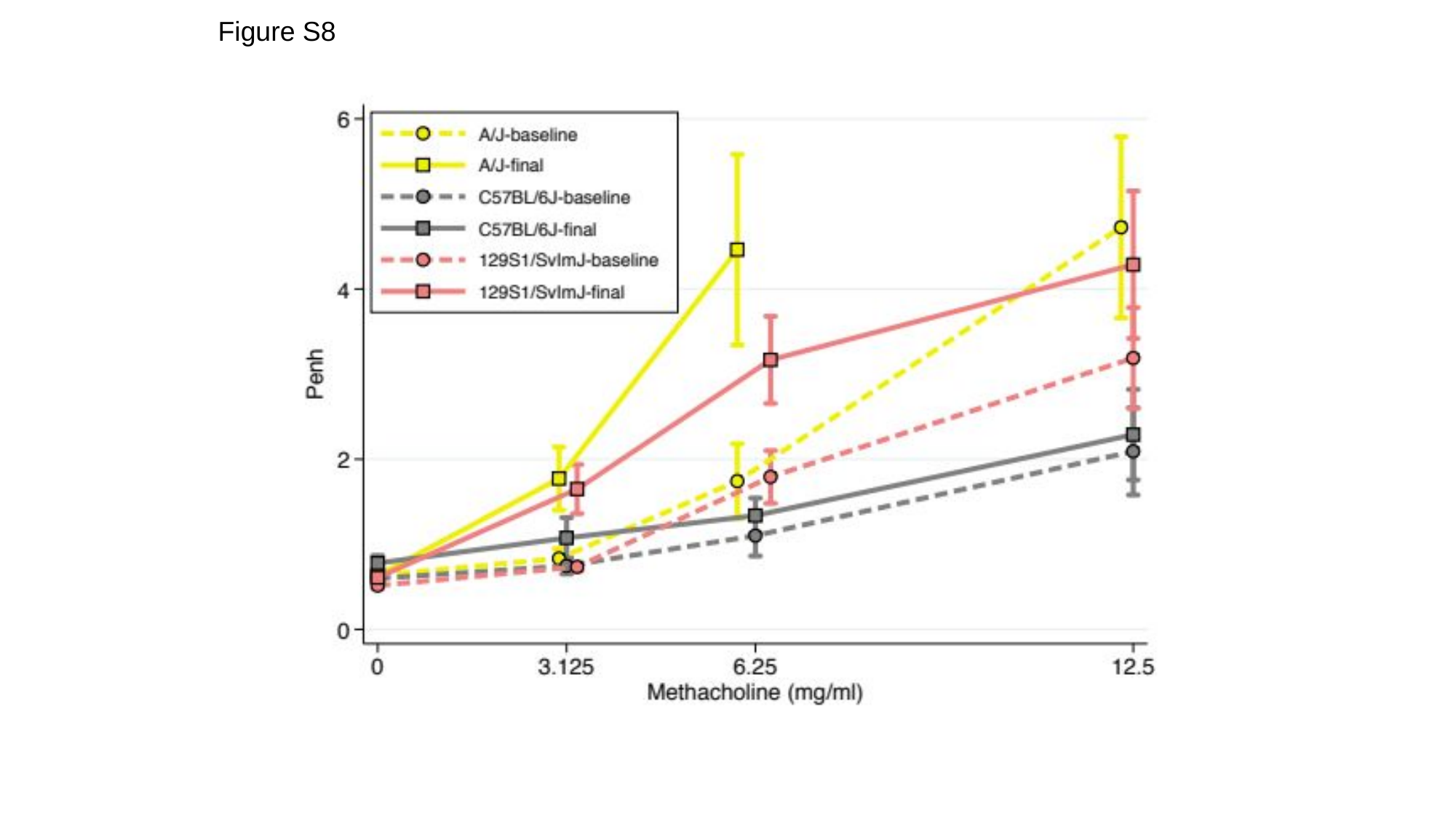

Figure S8

## Slide 2
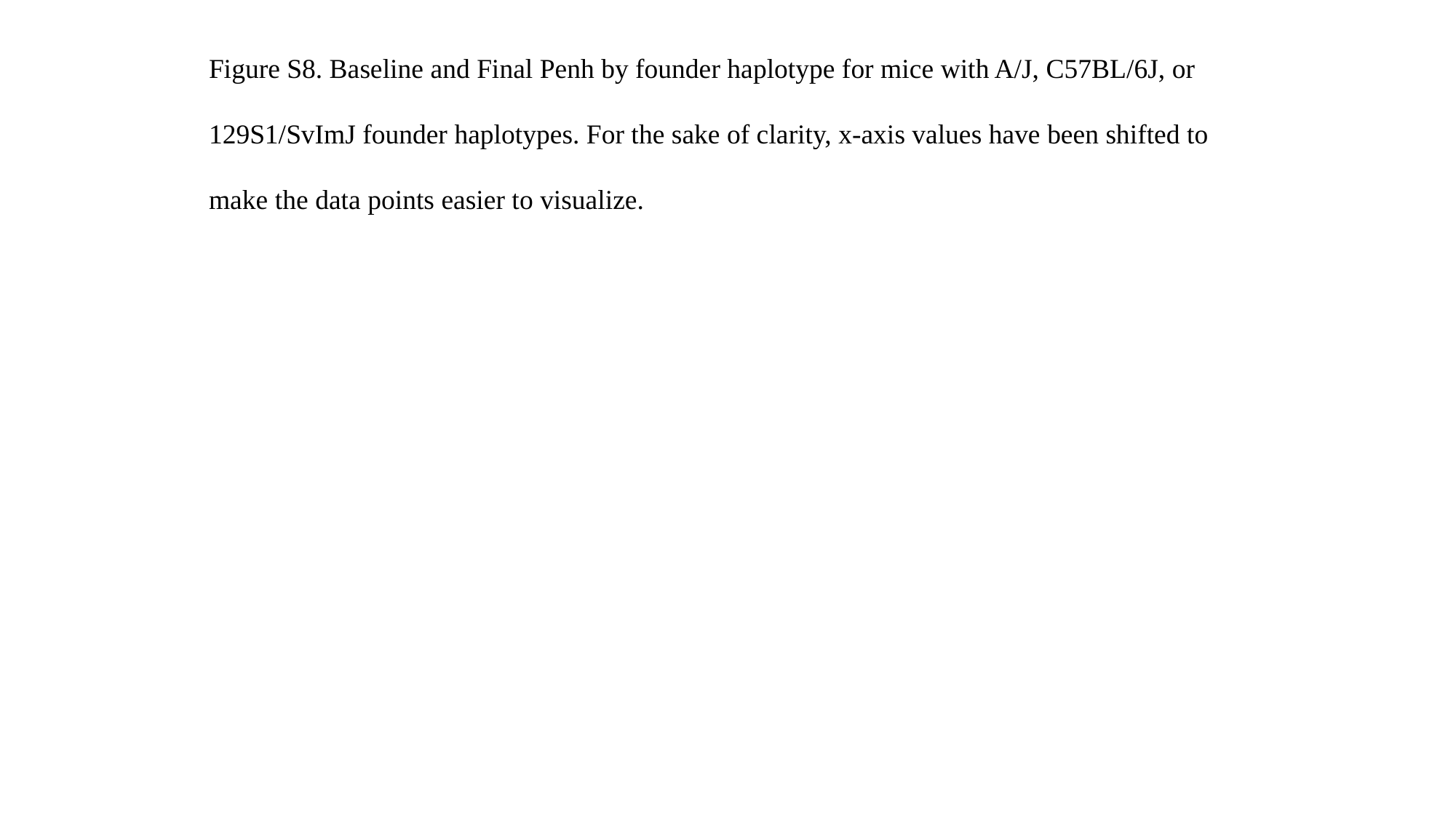

Figure S8. Baseline and Final Penh by founder haplotype for mice with A/J, C57BL/6J, or 129S1/SvImJ founder haplotypes. For the sake of clarity, x-axis values have been shifted to make the data points easier to visualize.
