# Supplementary material for: Plethysmography Phenotype QTL in Mice Before and After Allergen Sensitization and Challenge
Source: G3 (Bethesda). 2016 Jul 21;6(9):2857–65. doi: 10.1534/g3.116.032912 (PMC5015943; doi:10.1534/g3.116.032912)
Supplement: Supplemental Material [file supp_g3.116.032912_FigureS9.pptx]

## Slide 1
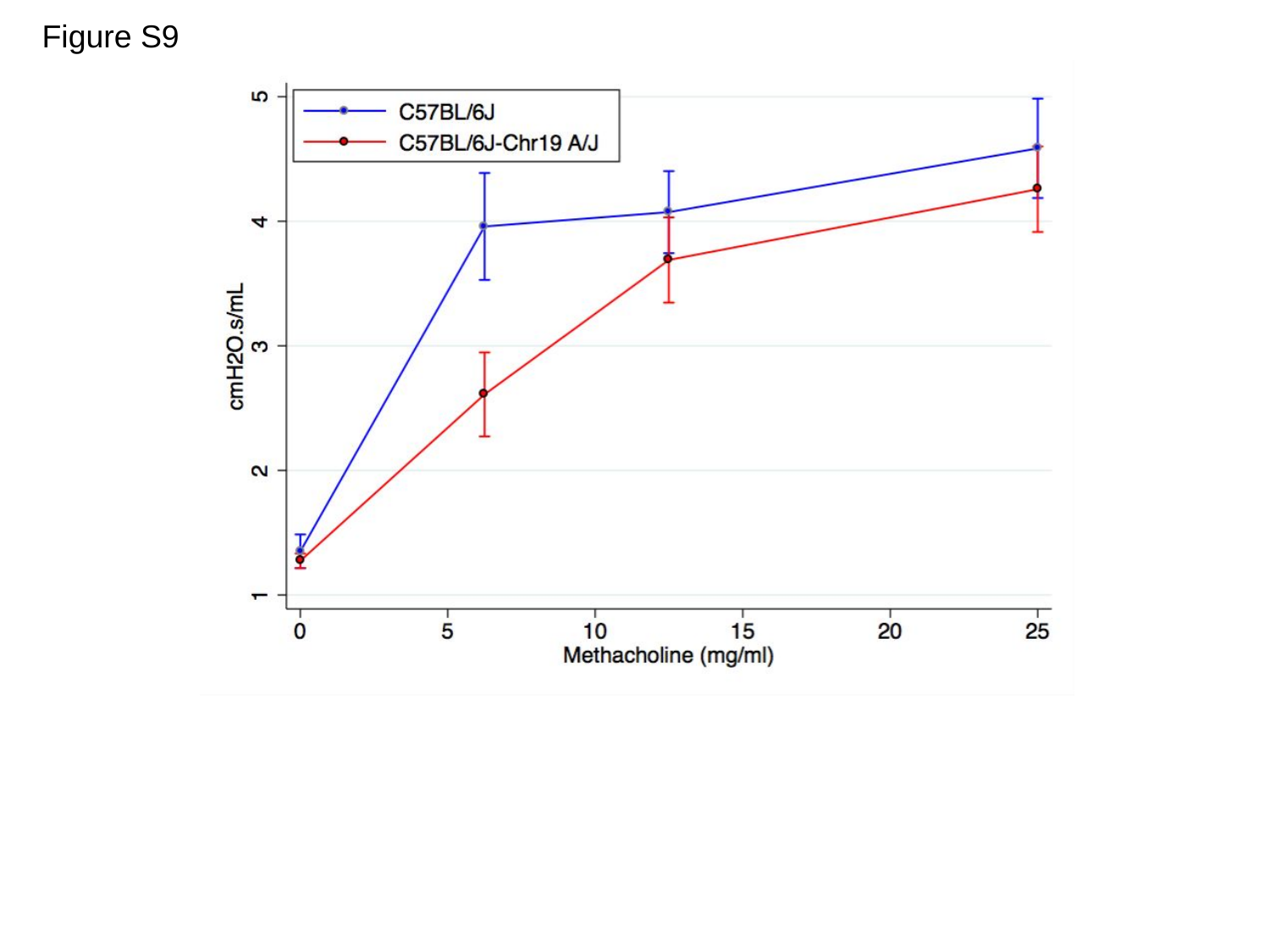

Figure S9

## Slide 2
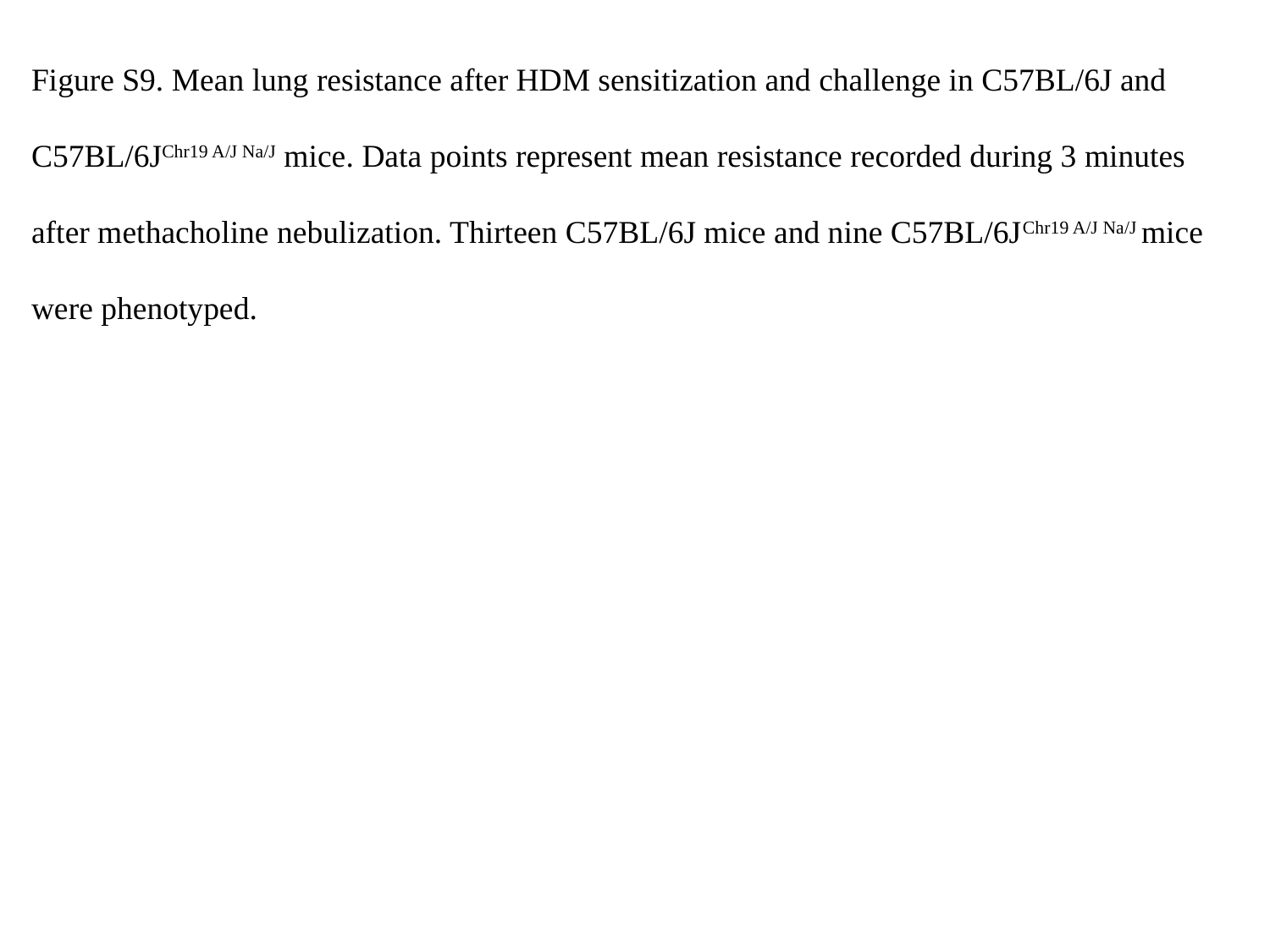

Figure S9. Mean lung resistance after HDM sensitization and challenge in C57BL/6J and C57BL/6JChr19 A/J Na/J mice. Data points represent mean resistance recorded during 3 minutes after methacholine nebulization. Thirteen C57BL/6J mice and nine C57BL/6JChr19 A/J Na/J mice were phenotyped.
